# Targeted resequencing reveals genomic signatures of barley domestication

**DOI:** 10.1111/nph.15077

**Published:** 2018-03-12

**Authors:** Artem Pankin, Janine Altmüller, Christian Becker, Maria von Korff

**Affiliations:** ^1^ Institute of Plant Genetics Heinrich‐Heine‐University 40225 Düsseldorf Germany; ^2^ Max Planck Institute for Plant Breeding Research 50829 Cologne Germany; ^3^ Cologne Center for Genomics (CCG) University of Cologne 50931 Cologne Germany; ^4^ Cluster of Excellence on Plant Sciences ‘From Complex Traits towards Synthetic Modules’ Heinrich‐Heine‐University 40225 Düsseldorf Germany

**Keywords:** ancestry analysis, barley, cereals, domestication, selection scan, targeted resequencing

## Abstract

- Barley (*Hordeum vulgare*) is an established model to study domestication of the Fertile Crescent cereals. Recent molecular data suggested that domesticated barley genomes consist of the ancestral blocks descending from multiple wild barley populations. However, the relationship between the mosaic ancestry patterns and the process of domestication itself remained unclear.
- To address this knowledge gap, we identified candidate domestication genes using selection scans based on targeted resequencing of 433 wild and domesticated barley accessions. We conducted phylogenetic, population structure, and ancestry analyses to investigate the origin of the domesticated barley haplotypes separately at the neutral and candidate domestication loci.
- We discovered multiple selective sweeps that occurred on all barley chromosomes during domestication in the background of several ancestral wild populations. The ancestry analyses demonstrated that, although the ancestral blocks of the domesticated barley genomes were descended from all over the Fertile Crescent, the candidate domestication loci originated specifically in its eastern and western parts.
- These findings provided the first molecular evidence implicating multiple wild or protodomesticated lineages in the process of barley domestication initiated in the Levantine and Zagros clusters of the origin of agriculture.

## Introduction

Domesticated barley (*Hordeum vulgare* L. ssp. *vulgare*) is one of the Neolithic founder crops, which facilitated establishment of the early agricultural societies (Lev‐Yadun *et al*., 2000). Owing to its striking environmental plasticity, barley is an important staple crop in a wide range of agricultural environments (Dawson *et al*., 2015). The first traces of barley cultivation were found at archaeological sites in the Fertile Crescent, which dated back to *c*. 10 000 bc (Zohary *et al*., 2012). The Fertile Crescent is the primary habitat of the crop progenitor wild barley (*H. vulgare* ssp. *spontaneum*). However, its isolated populations have spread as far as North African and European shores of the Mediterranean Basin and East Asia (Harlan & Zohary, 1966). Wild barley is a rich but under‐utilized reservoir of novel alleles for breeding of barley cultivars better adapted to predicted future climatic perturbations.

In contrast to some other crops, the visible phenotype of domesticated barley did not diverge dramatically from its wild form. So far, the spike rachis brittleness has remained the only well‐characterized domestication trait that exhibits a clear dimorphism between the wild and domesticated subgroups, which are characterized by the brittle and nonbrittle spikes, respectively (Abbo *et al*., 2014; Pourkheirandish *et al*., 2015). Other traits differentiated between the modern‐day wild and domesticated genotypes and underlying genes that define the barley domestication syndrome, as a complex of all characters that characterize the domesticated phenotype, are as yet undiscovered (Hammer, 1984; Meyer & Purugganan, 2013). When adaptive phenotypes are not clearly defined, the so‐called bottom‐up approach, which starts with the identification of genome‐wide signatures of selection, has proven instrumental in reconstructing the genetic architecture of the domestication syndrome (Ross‐Ibarra *et al*., 2007; Shi & Lai, 2015). In other crops, the selection scans detected multiple selective sweep regions associated with domestication, which comprised hundreds of candidate domestication genes (Huang *et al*., 2012; Hufford *et al*., 2012; Lin *et al*., 2014; Schmutz *et al*., 2014; Zhou *et al*., 2015).

The circumstances of barley domestication are debatable and its genome‐wide effects on the domesticated barley genomes remain poorly understood (Pankin & von Korff, 2017). The early models based on diversity analyses of isolated genes and neutral DNA markers proposed the Israel‐Jordan area as a primary center of cultivated barley origin and proposed the eastern Fertile Crescent, the Horn of Africa, Morocco and Tibet as the alternative centers of domestication (Negassa, 1985; Molina‐Cano *et al*., 1999; Badr *et al*., 2000; Morrell & Clegg, 2007; Dai *et al*., 2012). As regards the number and the timescale of domestication events, one school of thought maintains that Neolithic domestication in the Near East has been a rapid centric innovation (Abbo *et al*., 2010; Heun *et al*., 2012; Zohary *et al*., 2012). Conversely, the archeobotanical evidence and simulation studies prompted development of the so‐called protracted domestication model, which postulates that domestication of the Near Eastern crops has been a slow polyphyletic process dispersed over large territories (Allaby *et al*., 2008; Fuller *et al*., 2011, 2012; Purugganan & Fuller, 2011). The diphyletic origin of the nonbrittle spike phenotype of cultivated barley and the heterogeneous (mosaic) ancestry of cultivated barley genomes, which consisted of ancestral fragments originating from several wild barley populations, supported the protracted model (Allaby, 2015; Poets *et al*., 2015; Pourkheirandish *et al*., 2015). The heterogeneous origin of domesticated barley genomes hints at the existence of several founder lineages of barley cultivation. However, the link between the mosaic ancestry patterns and the process of domestication remained unclear.

Here, we partitioned the domesticated barley genomes into the neutral (in relation to domestication) and domestication sweep regions identified by selection scans and separately reconstructed the phylogeographic history of their origin, focusing on the hypothesis of the Fertile Crescent origins of domesticated barley. To this end, we resequenced a diversity panel comprising 344 wild barley accessions from the Fertile Crescent and 89 domesticated genotypes using a custom genome‐wide enrichment assay (*c*. 544 000 single nucleotide polymorphisms (SNPs)). The selection scans identified multiple domestication sweep regions on every barley chromosome. Analysis of the top candidate genes within the domestication sweeps suggested cases of parallelism in targets of selection during domestication of barley and other crop species. The patterns of ancestry at the neutral loci revealed signatures of abundant continuous gene flow, which hindered identification of lineages descending from the independent founder events. Nevertheless, heterogeneous ancestry of the domestication sweep loci provided the first molecular evidence that multiple domestication sweeps occurred in the background of several progenitor wild barley populations residing in the eastern and western clusters of the Fertile Crescent.

## Materials and Methods

### Plant material and Btr genotyping assay

A panel consisting of 344 wild and 89 domesticated lines was selected to maximize genetic diversity and to cover the entire range of the wild and landrace barley habitats in the Fertile Crescent (Supporting Information Table S1). The elite barley cultivars were sampled to represent northern European, East Asian, North American and Australian breeding programs. The largest part of the germplasm set, 98% of wild and 40% of domesticated barley genotypes, originated from the area of the Fertile Crescent. The selection of domesticated barley originated from various breeding programs and represented the whole variety of cultivated barley life forms, namely two‐ (71%) and six‐row (29%) genotypes with winter (45%) and spring (55%) growth habits based on the passport data. All material was purified by single‐seed descent to eliminate accession heterogeneity.

Leaf samples for DNA extraction were collected from single 3‐wk‐old plants. The DNA was extracted using the DNeasy Plant Mini kit (Qiagen) and quantified using the NanoDrop 1000 spectrophotometer (Thermo Fisher Scientific, Waltham, MA, USA) and electrophoresis in the 0.8% agarose gel.

The DNA samples of domesticated barley were genotyped using PCR markers distinguishing loss‐of‐function alleles of the brittleness genes *Btr1* and *Btr2*. The markers were amplified using allele‐specific primer pairs Btr1f 5′‐CCGCAATGGAAGGCGATG‐3′/Btr1r 5′‐CTATGAAACCGGAGAGGC‐3′ (*c*. 200 bp fragment, presence – *Btr1*/absence – *btr1*) and Btr2f 5′‐AATACGACTCACTATAGGGTTCGTCGAGCTCGCTATC‐3′/Btr2r 5′‐GTGGAGTTGCCACCTGTG‐3′ (*c*. 160 bp fragment, 11 bp deletion in the *btr2* allele). PCR reactions (1 × PCR buffer, 0.1 M primers, 1 U Taq polymerase, 100 ng DNA) were incubated in the PTC DNA Engine thermocycler (Bio‐Rad, Hercules, CA, USA) under the following conditions: 95°C for 3 min; 30 cycles of 95°C for 20 s, 60°C for 30 s, 72°C for 1 min; 72°C for 5 min.

### Design of the enrichment assay and SNP calling

To resequence barley genotypes, we designed a custom target enrichment assay, which included 666 loci implicated in the candidate domestication and environmental adaptation pathways in barley and other species and 1000 neutral loci covering all barley chromosomes to attenuate effects of the biased selection (Fig. S1; Table S2). Among the selected loci were known barley genes implicated in the regulation of flowering time, development of meristem and inflorescences, tillering, seed dormancy, and carbohydrate metabolism; barley homologs of flowering genes from the other grass species, such as Brachypodium and rice (Higgins *et al*., 2010); and barley homologs of 259 Arabidopsis genes characterized by the development‐related gene ontology (GO) terms that have been confirmed experimentally (Table S3). The barley homologs were extracted from the following sources: the NCBI UniGene dataset (ftp://ftp.ncbi.nih.gov/repository/UniGene/Hordeum_vulgare), IBGSC High and Low confidence genes (IBGSC, 2012), and the HarvEST unigene assembly 35 (http://harvest.ucr.edu). See Methods S1 for the detailed description of the gene selection and design of the capture baits.

The SNP calling pipeline consisted of three modules: quality control and filtering of Illumina read libraries; mapping the reads to the custom reference (Methods S2; Table S4); and extracting and filtering both variant (SNP) and invariant sites (Fig. S2), implemented in a series of bash scripts using standard bioinformatics tools (Methods S3). Genetic positions of the reference contigs were determined according to two versions of the POPSEQ maps, which are hereafter termed Mascher_2013 (Morex × Barke map; Mascher *et al*., 2013b) and Beier_2017 (Beier *et al*., 2017). Homology between the selected target loci and HORVU genes (Mascher *et al*., 2017) was determined using reciprocal blastn algorithm.

To determine the ancestral status, the SNPs were genotyped *in silico* in two *Hordeum* species, *H. bulbosum* and *H. pubiflorum*, using the Hordeum exome Illumina datasets and the aforementioned bioinformatics pipeline (Mascher *et al*., 2013a). Alleles that were identical in both species were assigned as ancestral.

*De facto* captured regions were defined as those with the depth of coverage ≥ 8 in at least one of the samples, which was determined using bedtools v.2.16.2, vcftools v.0.1.11 and R (Danecek *et al*., 2011; Quinlan, 2014). Functional effects of the SNPs were predicted using snpeff 3.6b with the custom CDS coordinates (Cingolani *et al*., 2012). The coordinates were determined on the target genomic contigs based on the spidey predictions (Wheelan *et al*., 2001).

### Population structure

The population structure was explored using Bayesian clustering algorithms (structure and instruct), maximum‐likelihood (ML) and Neighbor‐Net phylogenetic analyses and principal component analysis (PCA). For all the population structure analyses, the subset of putatively neutral SNPs based on the snpeff flags with minor allele frequency > 0.05 and missing data frequency < 0.5 was selected. The vcf files were converted into the ped format using the tabix utility of samtools and plink 1.9 (Chang *et al*., 2015). The SNPs in very high linkage disequilibrium (*r* ^2^ > 0.99) were pruned using plink. The PCA was performed using the smartpca utility of the eigensoft software 5.0.2 (Patterson *et al*., 2006).

The faststructure software (Raj *et al*., 2014) was applied with 20 iterations for a predefined number of populations (*K*). The optimal *K* for wild barley was chosen to represent the model with maximum marginal likelihood tested for *K* from 2 to 25. The output matrices were summarized using clumpak (Kopelman *et al*., 2015), reordered and plotted using an in‐house R script. The instruct tools, which extends the structure model to include selfing (Gao *et al*., 2007), owing to very high computational intensity, was run on 10 randomly drawn subsamples of 1000 SNP markers for five independent chains.

The geographic centers of the populations were calculated as a median of the latitude and longitude of the genotypes comprising the populations. The vector geographic map dataset was downloaded from Natural Earth repository and manipulated in R (http:/www.naturalearthdata.com).

The ML phylogeny rooted to *H. bulbosum* and *H. pubiflorum* outgroup species was estimated from the genome‐wide SNP dataset using the GTRCAT model with Lewis's ascertainment bias correction to account for the absence of invariant sites in the alignment and the rapid bootstrap search, which stopped after 200 replicates according to the majority‐rule tree‐based criteria for bootstopping (autoMRE_IGN) implemented in RAxML 8.2.8 (Stamatakis, 2014). The admixed wild genotypes (faststructure single population ancestry component < 95%) were excluded from the input dataset as gene flow between the genotypes may lead to inaccurate placement of the admixed accession on the bifurcating phylogenetic tree. The trees were manipulated using dendroscope 3.5.7 (Huson & Scornavacca, 2012). The Neighbor‐Net phylogenetic network was constructed for all the wild and domesticated accessions using splitstree 4.14.6 (Huson & Bryant, 2006).

### Identification of domestication sweeps

The putative signatures of selection related to domestication were identified using several complementary tests – the diversity reduction index (*π*_wild_/*π*_domesticated_, DRI), Fay&Wu's *H*_norm_ (Zeng *et al*., 2006) and the composite likelihood ratio (CLR) test. The *π* and *H*_norm_ statistics were calculated for the individual loci and sliding 10 cM windows (step 1 cM) using mstatspop software, which account for missing genotypes in the data, with 1000 permutations (release 0.1b 20150803; http://bioinformatics.cragenomica.es/numgenomics/people/sebas/software/software.html). A sum of segregating and invariant sites was used to normalize the *π*‐values. The software sweeD 3.3.2 (Pavlidis *et al*., 2013) was used to calculate the CLR test of Kim & Stephan (2002) as expanded by Nielsen *et al*. (2005). sweeD calculates the ratio of the maximum composite likelihoods under a neutral model, which uses the genome‐wide site frequency spectrum (SFS) as a reference, to the maximum composite likelihoods under a selective sweep model. The CLR tests were calculated separately for wild and domesticated subsets from the unfolded SFS of individual reference contigs containing at least four SNPs at two grid points across each contig. The genome‐wide reference SFS was calculated using sweeD's ‘‐osfs’ flag and provided for the CLR calculations for the individual loci.

To estimate statistical thresholds of the CLR neutral distribution, following Nielsen *et al*. (2005), we simulated 1000 datasets assuming a standard neutral model without recombination using the coalescent simulation software ms with the number of segregating sites (S) and the number of samples (*n*) as the input parameters describing the wild and domesticated barley populations (Hudson, 2002). Variation of the CLR and in the simulated neutral datasets was assessed using sweeD with the threshold to reject neutrality at the 99^th^ percentile of the neutral CLR values. The variation of *H*_norm_ in the same neutral datasets was assessed using the msstats software (https://github.com/molpopgen/msstats) and the threshold was chosen as the 99.9^th^ percentile to minimize the number of false positives as a result of the likely deviation of barley demographic history from the standard model. For the DRI, the top 95^th^ percentile was used as a cutoff value following Liu *et al*. (2017). Significance of the overlaps between the tests was estimated using hypergeometric test in R.

### Ancestry of domesticated barley genomes

To estimate ancestry of the domesticated barley loci, we calculated pairwise ML distances between each wild and domesticated genotype separately for each locus (i.e. individual contig in the mapping reference; in total 39.6 million comparisons) using the GTRGAMMA model in RAxML 8.2.8 (Stamatakis, 2014). If an allele in a domesticated genotype had a smallest ML distance with a population‐specific wild allele, this wild allele was deemed ancestral for this locus in this domesticated genotype. The cases where a domesticated barley allele was equally distant to wild barley alleles found in several populations represent the instances of incomplete lineage sorting and therefore were not accounted for in a cumulative ancestry of a genotype. A sum of all loci with assigned origin in a single domesticated accession sorted by the locus name we termed an ‘ancestry palette’. The ancestry palettes of the individual accessions were pairwise‐correlated using the Jaccard index‐based similarity measure (*J*) implemented in R:

$$J\left(X,Y\right)=\frac{\left|X⋂Y\right|}{\left|X|+|Y|+|X⋂Y\right|}$$

where *X* and *Y* are vectors of individual elements of the ancestry palettes, i.e. concatenated locus name and corresponding ancestral population (e.g. locus1_population1) in a pair of genotypes. The ancestry similarity of 1 means identical ancestry palettes and that of 0 means that no loci originate from the same population in a pair of accessions. Ancestry similarity heatmaps were visualized using ‘heatmap.2’ function of the ‘gplots’ R package.

The kernel density estimates were plotted using the ‘geom_density’ function of the ggplot2 R package with the values binned into the 0.1× and 2× of the default bin size for the distributions of the longitudes and the similarity indices, respectively.

In this study, we used the term ‘protodomesticated lineage’ to describe descendants of an event when barley cultivation has been initiated from a wild barley population, in which at least one of the selective sweeps found in the modern‐day cultivated barley genomes (detected in this study) occurred. The scripts and the accompanying files used for the analyses are available in an online repository at https://github.com/artempankin/korffgroup.

## Results

### More than 500 000 SNPs discovered by the targeted resequencing assay

A total of 433 barley accessions, including 344 wild and 89 domesticated barley genotypes, were analyzed in this study. To maximize diversity, the barley genotypes were selected to cover the entire range of wild barley habitats in the Fertile Crescent and to represent the whole variety of domesticated barley life forms from various breeding programs (Table S1). Additionally, domesticated barley is classified into the *btr1* (*btr1Btr2*) and *btr2* (*Btr1btr2*) types based on the allelic status of the spike brittleness genes *Btr1* and *2*; independent mutations in either of these genes convert the wild‐type brittle spikes into the nonbrittle spikes of the domesticated forms (Pourkheirandish *et al*., 2015). To further verify representativeness of the selected genotypes, we screened for the *Btr* mutations using allele‐specific markers. In our genotype set, the *btr1* and *btr2* types were represented by 71% and 29% of the domesticated accessions, respectively (Table S1).

Illumina enrichment resequencing of 433 barley genotypes yielded *c*. 8 billion reads (0.56 Tb of data; deposited at NCBI SRA BioProject PRJNA329198; Notes S1). Cumulatively, the captured regions comprised *c*. 13.8 Mbp (Table S5), 1.33 Mbp of which resided in the coding regions (CDS). Per‐sample analysis of the coverage revealed that *c*. 87% of the captured regions were covered above the SNP calling threshold and that the between‐sample variation was relatively low, with the median depth of coverage varying from 45 to 130 (Fig. S3). The SNP calling pipeline identified 544 318 high‐quality SNPs, including *c*. 190 000 singletons (Table S5). Of all the SNPs, 37 870 resided in CDS and *c*. 43% of them were nonneutral based on the Snpeff predictions. The CDS were more conserved than the noncoding regions with the average SNP density of 29 and 41 SNPs per kbp, respectively; 45% of the SNPs could be located on the barley genetic map, whereas for 37% of the SNPs, only the chromosome could be assigned (Fig. S4).

### Patterns of recent admixture between wild and domesticated barley

**Figure 1:**
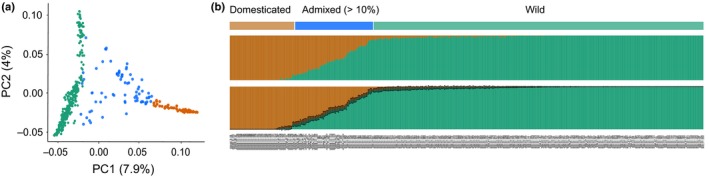
Genome‐wide analysis of admixture between wild and domesticated barley. (a) Principal component (PC) analysis of 433 barley genotypes. The first two PCs discern subgroups of wild (green) and domesticated (orange) barley and admixed (blue) genotypes. A percentage of the total variation explained by the PCs is shown in parentheses. (b) Global genetic ancestry of the wild and domesticated barley genotypes as determined by the population structure analysis using faststructure (*c*. 315 000 SNPs) and instruct (10 random samples of 1000 SNPs) models – upper and lower panels, respectively. Proportions of wild and domesticated ancestral clusters are shown as green and orange vertical bars, respectively. The standard deviations on the instruct plots are shown as whiskers.

In domestication studies, where patterns of genetic variation are contrasted between wild and domesticated genotypes, it is critical to distinguish these subgroups and exclude genotypes of unclear provenance. The PCA based on the SNP markers revealed two distinct clusters corresponding to the domesticated and wild subspecies, with multiple genotypes scattered between these clusters (Fig. 1a). faststructure analysis (*K* = 2) revealed patterns of recent admixture between wild and domesticated subspecies in 36% and 12% of the domesticated and wild genotypes, respectively. These admixed accessions corresponded to the genotypes intermediate between wild and cultivated barley clusters in the PCA (Fig. 1b). Both faststructure and instruct models produced matching admixture patterns (*r* ^2^ > 0.99) (Fig. S5). The admixed domesticates did not originate from any specific locality and the admixed wild barley were spread all over the Fertile Crescent, indicating that the admixture was not restricted to any particular geographical area (Table S1). These admixed genotypes of ambiguous provenance were removed from further analyses.

### Footprints of domestication‐related selection

Selection acting on a beneficial mutation affects various aspects of genetic variation such as allele frequency, nucleotide diversity and linkage disequilibrium in the neighboring regions in a process called selective sweep. To scan for signatures of selective sweeps, which occurred during domestication (hereafter domestication sweep), we performed genome scans using several statistics – the CLR, Fay & Wu's *H*_norm_, and the DRI (*π*_wild_/*π*_domesticated_). These statistics explore different patterns of molecular variation and therefore presumably reveal signatures left by selection under different scenarios (Innan and Kim 2004). Both the CLR and *H*_norm_ statistics are site frequency‐based and describe variation of the SFS at the tested loci. The *H*_norm_ statistics detects enrichment of the high‐frequency derived alleles (the right tail of an unfolded SFS), whereas sweeD CLR takes advantage of the patterns of variability in the complete SFS scanned along the sequence length. Strong deviations of these statistics from the expected genome‐wide values, as tested by coalescent simulations under the neutral scenario, indicate selection. The DRI statistics reveals a severe depletion of nucleotide diversity in the domesticated genotypes at certain loci detected as statistical outliers. All three statistics have frequently been used in the selection scans to reveal domestication sweeps and candidate domestication genes in other crop species (Huang *et al*., 2012; Lin *et al*., 2014; Wang *et al*., 2014; Civáň *et al*., 2015; Velasco *et al*., 2016; Zhong *et al*., 2017).

**Figure 2:**
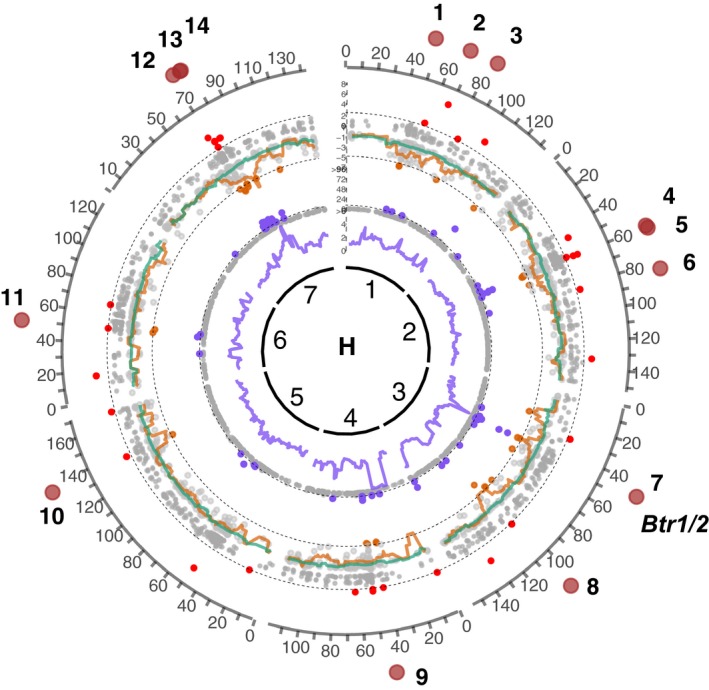
Genomic signatures of domestication selective sweeps (Beier*_*2017 map). Genome scans for signatures of selection associated with domestication. The sliding‐window and individual‐target values are shown as lines and points, respectively. The innermost circle represents barley linkage groups (*H*) followed by the diversity reduction index (*π*_wild_/*π*_dom_) (violet); the normalized Fay&Wu's *H*_norm_ statistics for the wild (green) and domesticated (orange) groups; and the composite likelihood ratio statistics (sweeD CLR) for the domesticated group (red). The outlier thresholds are shown by dashed lines and the nonoutlier loci are shown as gray dots for all the tests. Fourteen candidate selected regions supported by at least two of the statistics are shown as brown circles on the outermost layer. *Btr1*/*2*, brittle rachis domestication genes (Pourkheirandish *et al*., 2015).

Altogether the scans identified 137 outlier contigs carrying signatures of a selective sweep – 91 of them could be located on the map and covered all barley chromosomes (Table S6). Of those, 20 contigs (16 and 14 mapped locations on Mascher_2013b and Beier*_*2017 maps, respectively) were outliers in at least two of the scans (Figs 2, S6; Table S6). The overlap between the CLR and *H*_norm_ scans was relatively high, 38% (*P*‐value < 1.0e–07), and the overlaps between the DRI scan and the other two tests were significant but less prominent (8–10%; *P*‐value < 0.05) consistent with the previously reported values (Liu *et al*., 2017).

Among the top outliers including the loci in the overlaps between the tests were homologs of genes implicated in the modulation of the light signaling, circadian clock, and carbohydrate metabolism pathways (Table S6). None of the candidate domestication genes identified in this study have been functionally characterized in barley; however, putative function can often be inferred from homology.

In our scans, the barley homolog of the Arabidopsis gene *EMPFINDLICHER IM DUNKELROTEN LICHT 1 – like 3* (*EDL3*) of the *EID1* gene family had the strongest CLR signal among all the genes (seq375; CLR = 7.13). In Arabidopsis, *EDL3* has been implicated in regulation of the photoperiod pathway and ABA signaling, whereas in tobacco, the *EDL3* homolog encodes a key element of the circadian clock (Koops *et al*., 2011; Xu and Johnson, 2001). In tomato, a circadian clock gene homologous to *EID1* has been implicated in domestication (Müller *et al*., 2016). The homologs of the Arabidopsis *CULLIN4* (*CUL4*; seq442, AK371672; *H*_norm_ = −5.1, CLR = 2.7) and *SUPRESSOR OF PHYA 2* (*SPA2*; seq108, MLOC_52815; DRI = 53), encoding two members of the COP1‐CUL4‐SPA protein complex implicated in regulation of the light signaling pathway, are other examples where strong signatures of selection have been found in our scans and in another crop species (Zhu *et al*., 2008). In the common bean, the homologs of *CUL4* and *CONSTITUTIVELY PHOTOMORPHOGENIC 1* (*COP1*) – a gene encoding another member of the COP1‐CUL4‐SPA complex – have been independently targeted by selection in two separate domestication events (Schmutz *et al*., 2014). Two genes of the starch metabolism pathway – the homologs of the alpha‐ and beta‐amylases (AMY, seq669, DRI = 23; BAM1, seq345, *H*_norm_ = −5.32) – were strong outliers in the DRI and *H*_norm_ scans. Previous studies discovered reduced variation at the alpha‐amylase locus in barley domesticates compared with wild genotypes and hinted at the functional divergence of the wild and domesticated alpha‐amylase alleles (Kilian *et al*., 2006; Cu *et al*., 2013).

The location of the only known barley domestication locus *Btr1/2* coincided with the domestication sweep 6 on the chromosome 3 and thus the *Btr1/2* genes were probably direct targets of selection within the sweep 6 (Fig. 2). The location of selective sweeps 13–16 presumably corresponded to the region of depleted diversity on the chromosome 7 discovered by Russell *et al*. (2016), who speculated that the *NUD* gene, controlling the naked (hulless) grain phenotype (Taketa *et al*., 2004), might have been a direct target of domestication in this region. In our scans, the *NUD* gene itself did not carry a selection signature and thus apparently was not the target of selection at this locus. Indeed, both hulless and hulled genotypes are ubiquitously present in the domesticated barley gene pool and thus the naked grain phenotype represents an improvement but not a domestication trait (Saisho & Purugganan, 2007).

### Population structure and ancestry analyses

Next, we investigated whether the local ancestry patterns in cultivated barley genomes could shed light on the phylogeographic history of barley domestication. To this end, we first explored the population structure of wild barley genotypes using Bayesian clustering, PCA and phylogenetic analyses and then searched within the wild gene pool for putative ancestral alleles for each locus of the domesticated genotypes using the ML approach (see the Materials and Methods section; Fig. S7). Following a widely held assumption, we assumed that any individual genomic locus in a domesticated barley accession descended from a wild population that carried the phylogenetically closest allele and that the habitat of this wild population indicates a place of origin of the cultivated barley allele (Civáň *et al*., 2015; Pourkheirandish *et al*., 2015). The cases where a putative ancestral allele was not specific to a single wild population were excluded from the analysis to alleviate adverse effects of the incomplete lineage sorting on the ancestry estimates.

**Figure 3:**
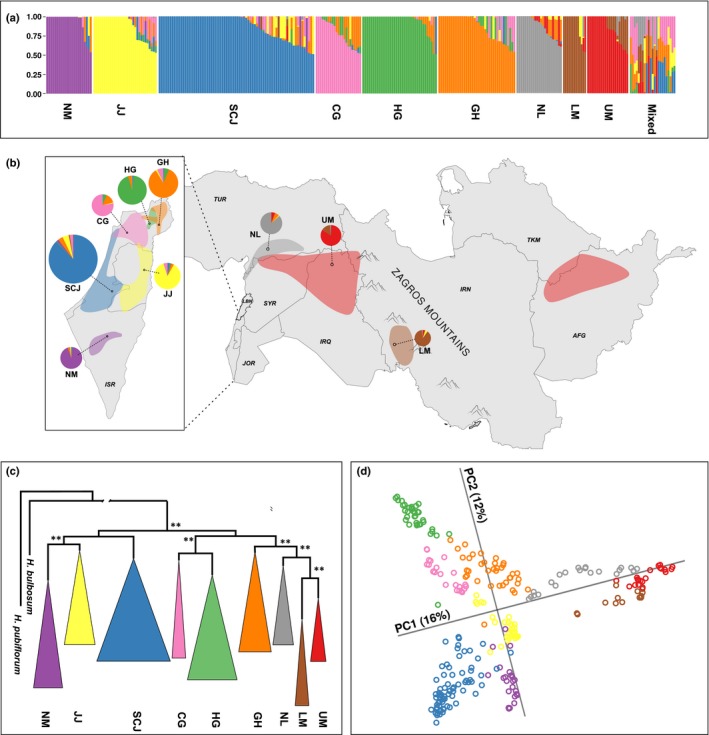
Geographic distribution, structure and phylogeny of nine wild barley (*Hordeum vulgare* ssp. *spontaneum*) populations. The colors correspond to the nine wild barley populations: Carmel & Galilee (CG, pink); Golan Heights (GH, orange); Hula Valley & Galilee (HG, green); Judean Desert & Jordan Valley (JJ, yellow); Lower Mesopotamia (LM, brown); Negev Mountains (NM, magenta); North Levant (NL, gray); Sharon, Coastal Plain & Judean Lowlands (SCJ, blue); and Upper Mesopotamia (UM, red). (a) Population structure of wild barley as determined by faststructure for *K* = 9. Vertical bars correspond to individual genotypes and colors indicate their membership in the nine subpopulations. (b) Distribution of the wild barley populations within the Fertile Crescent. The pie charts represent the ancestral composition of the populations as determined by faststructure and are connected to the geographic centers of population distributions by dashed lines. The size of the pie charts reflects the number of genotypes in the populations. The country codes (ISO 3166) are shown in italics. (c) The maximum likelihood phylogeny of wild barley accessions (faststructure single population ancestry component > 95%). The clusters were collapsed based on the population assignment. *Hordeum bulbosum* and *Hordeum pubiflorum* were used as distant outgroup species and the length of the outgroup branch was artificially shortened. The branches with the bootstrap support values > 0.6 are labeled by double asterisks. (d) Principal component (PC) analysis of wild barley accessions (faststructure single population ancestry component > 95%). Only first two PCs are shown. A percentage of the total variation explained by the PCs is shown in parentheses.

Nine wild barley populations were suggested by faststructure, which corresponded to the clearly defined clusters on the ML phylogenetic tree and the Neighbor‐Net network (Figs 3abc, S8, S9, S10; Notes S2). The domesticated barley genotypes branched off as a monophyletic cluster on the ML phylogram at a sister position to the cluster of wild barley genotypes (Fig. S9). This apparently inaccurate position of the domesticated barley cluster on the ML phylogeny indicated a complex reticulate genealogy of the domesticated barley genomes that could not be reliably described by a bifurcating tree.

Six wild populations (Carmel and Galilee (CG), Golan Heights (GH), Hula Valley and Galilee (HG), Judean Desert and Jordan Valley (JJ), Negev Mountains (NM), Sharon, Coastal Plain and Judean Lowlands (SCJ)) were concentrated in the South Levant and the other three (Lower Mesopotamia (LM), North Levant (NL) and Upper Mesopotamia (UM)) occupied large areas of the northern and eastern Fertile Crescent. Habitats of the wild populations were distinct, with very few immigrants and genotypes of mixed ancestry occurring mostly in the borders of overlapping areas (Figs S11, S12). Only 23 wild accessions had a highly admixed ancestry and could not be attributed to any of the nine populations (Fig. 3a).

Principal component analysis of wild barley genotypes identified nine clusters, which were significantly different between each other (pairwise *P*‐values < 1e–55) and corresponded to the wild barley populations defined by faststructure (Fig. 3d; Table S7). The patterns of genetic diversity revealed by PCA mirrored the patterns of geographic distribution of the wild barley populations within the Fertile Crescent.

**Figure 4:**
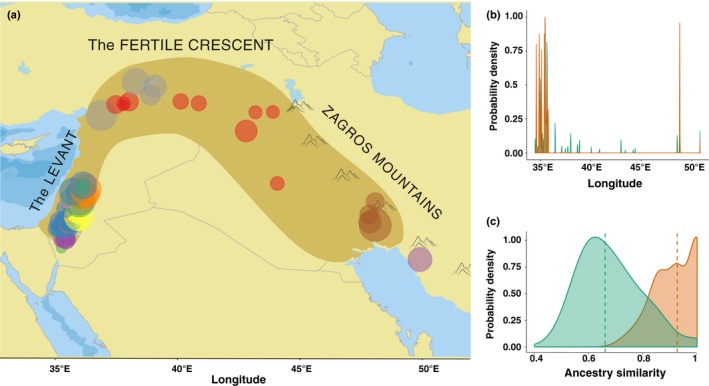
Origin and ancestral composition of the domesticated barley genomes. (a) Geographic distribution of wild barley accessions, which carry ancestral haplotypes of the domesticated barley (*Hordeum vulgare* ssp. *vulgare*) loci, within the Fertile Crescent. The colors correspond to the nine wild barley (*H. vulgare* ssp. *spontaneum*) populations: Carmel & Galilee (pink); Golan Heights (orange); Hula Valley & Galilee (green); Judean Desert & Jordan Valley (yellow); Lower Mesopotamia (brown); Negev Mountains (magenta); North Levant (gray); Sharon, Coastal Plain & Judean Lowlands (blue); and Upper Mesopotamia (red). (b) Longitudinal distribution of the ancestral wild haplotypes for the neutral (green) and domestication sweep loci (orange) shown as kernel density plots. (c) Distribution of the pairwise ancestry similarity coefficients estimated for the neutral (green) and domestication sweep loci (orange) shown as kernel density plots. The vertical dashed lines indicate the median similarity values for the corresponding subgroups.

A putative wild ancestor could be assigned for 1232 loci separately in each domesticated genotype (Fig. 4a). In all, 60% of the loci were monophyletic, i.e. descended from the same wild barley population in all domesticated genotypes, whereas ancestry of 40% of the loci could be traced back to several wild populations across the domesticated genotypes. For further analyses, we separated the dataset into two parts: the candidate domestication loci, to identify geographical origin of the domestication sweep events even without knowing direct targets of selection; and the rest of the genome, which we tentatively termed neutral, to search for the genome‐wide signatures of independent founder lineages.

The geographical distribution of the ancestral haplotypes of the neutral loci suggested that wild barley populations from every sampled part of the Fertile Crescent contributed to the domesticated genomes (Fig. 4a). In contrast to the neutral loci, the longitudinal distribution of the haplotypes ancestral to the domestication sweep loci was significantly different (Kolmogorov–Smirnov test, *P*‐value < 1e–12) (Fig. 4b). The domestication sweep loci descended from two discernible clusters of wild barley genotypes in the eastern and western parts of the Fertile Crescent (Fig. 4b). Intriguingly, the proportions of individual contributions of the ancestral wild populations did not differ noticeably between the domesticated genotypes, hinting at a single, highly admixed progenitor lineage at the root of domesticated barley (Fig. S13).

To gather further evidence on this hypothesis, we quantified the similarity of the ancestry patterns in the genomes of the domesticates by pairwise correlation of the sorted ancestry palettes of individual accessions (Fig. S14). The ancestry palettes of the neutral loci were only moderately similar (median 0.64), which means that, for multiple loci, the patterns of ancestry were not consistent across the domesticated genotypes (Fig. 4c). Apparently this dissimilarity of the ancestral patterns at multiple loci resulted from a gene flow, which randomly shuffled alleles descending from different ancestral wild populations between the domesticated genotypes. By contrast, the ancestry palettes of the domestication sweep loci were remarkably similar (median 0.96) across the domesticated genotypes compared with the neutral genome, indicating that the randomizing effect of gene flow was considerably weaker at the genomic regions maintaining the domestication syndrome (Fig. 4c). It is noteworthy that this difference between the similarity of the ancestry palettes in the neutral and domestication sweep loci did not arise from the unbalanced number of loci in the subgroups (Fig. S15). In both subsets, the heatmaps of the ancestry similarity did not reveal any clear‐cut patterns, e.g. presence of several discernible clusters, which could be interpreted as a signal of distinct founder lineages (Fig. S16).

Several domestication models may explain the discovered ancestry patterns. One of the candidate scenarios implicates independent protodomesticated lineages, which originated from several founder events in the eastern and western parts of the Fertile Crescent. These lineages could have been combined by means of gene flow into a single admixed progenitor lineage, which was at the root of the domesticated barley genotypes (Fig. S17). An alternative hypothesis suggests that a single founder lineage of domestication may have experienced gene flow from the wild populations and that the selective sweeps occurred sequentially in the background of the ancestral fragments of heterogeneous origins (Fig. S18). In both cases, continuing gene flow between wild and domesticated subspecies further randomized the ancestry patterns of the modern domesticated genotypes, particularly at the selectively neutral loci.

## Discussion

### Selection scans reveal multiple domestication sweeps in barley

Our understanding of the genes and traits that constitute the barley domestication syndrome is extremely limited. Here, the selection scans identified a domestication sweep at the *Btr1/2* locus, which modulates spike brittleness – the only studied example of a crucial domestication trait (*sensu* Abbo *et al*., 2014). We also discovered multiple novel candidate domestication genes, which are implicated in the regulation of light signaling, circadian clock, and carbohydrate metabolism pathways. It is noteworthy that the domestication loci detected in this study may be only a representative sample of the truly selected loci. Some loci might have experienced selection regimes leaving signatures that escape detection, confounded with the effects of demography, or that are missed because of the gaps in certain regions of the genetic and physical maps (Teshima *et al*., 2006; Mascher *et al*., 2013b, 2017; Beier *et al*., 2017).

Intriguingly, we found examples of genes carrying selection signatures in both barley and other crops, suggesting convergence of domestication‐related selection on homologous developmental pathways and protein complexes in different crop species. The most prominent examples were a circadian clock gene of the *EID1* family, the homolog of which is a domestication gene in tomato (Müller *et al*., 2016), and genes SPA and CUL4 encoding components of the E3 ubiquitin‐ligase COP1‐CUL4‐SPA, which was targeted by domestication of common bean. The COP1‐CUL4‐SPA complex is a critical part of the far‐red light signaling, photoperiod and circadian clock pathways (Zhu *et al*., 2015).

This finding adds to the growing evidence that components of the circadian clock, light signaling and shade‐avoidance pathways were targets of adaptive selection during domestication or further adaptation to new agronomic environments in various crop species (Faure *et al*., 2012; Zakhrabekova *et al*., 2012; Müller *et al*., 2016; Shor & Green, 2016). However, the evolutionary role played by such modifications in the domestication syndrome has not been understood. Müller *et al*. (2016) suggested that modification of the circadian clock was a human‐mediated adaptation of cultivated tomato, which was domesticated in the equatorial regions, to long photoperiods of the northern latitudes. In our study, many of the domesticated barley genotypes originated from the same latitude where barley domestication ensued, which makes, in the case of barley, the scenario of adaptation to a latitudinal cline less plausible. An alternative hypothesis might be linked to the fact that many crop plants are cultivated in dense stands that result in dramatic changes in the light environment and, as a consequence, alter plant architecture compared with their wild ancestor species. Therefore, we propose that such common patterns in the crop adaptation to agricultural practices might be the key to understanding the involvement of the modulators of light signaling, circadian clock and shade‐avoidance pathways in domestication.

### Ancestry of the candidate domestication loci relates the mosaic model and the process of domestication

Identification of the candidate domestication genes enables the phylogeorgaphic origin of the domestication sweep events to be predicted, which, together with the surveys using neutral markers revealing the closest wild ancestor of the domesticated populations at the genome‐wide level, represent two complementary approaches to untangling domestication histories (Badr *et al*., 2000; Matsuoka *et al*., 2002; Morrell & Clegg, 2007; Huang *et al*., 2012; Civáň *et al*., 2015; Poets *et al*., 2015; Pourkheirandish *et al*., 2015). Here, the heterogeneous ancestry of the candidate domestication loci provided compelling evidence that multiple domestication sweeps occurred in the background of various founder populations of wild barley. The ancestral populations of the domestication sweep loci were confined to the eastern and western parts of the Fertile Crescent.

The dominant narrative of the barley domestication history has long since revolved around the idea of the two independent domesticated lineages originating in the Levantine (west) and Zagros (east) horns of the Fertile Crescent (hereafter east–west model). It stems from the finding suggesting the existence of the Occidental and Oriental types of domesticated barley corresponding to the *btr1* and *btr2* types, respectively (Takahashi, 1955). Later, the east–west model was supported by molecular analyses of the barley population structure, as well as by archaeological studies (Azhaguvel & Komatsuda, 2007; Morrell & Clegg, 2007; Riehl *et al*., 2012, 2013; Tanno & Willcox, 2012; Fang *et al*., 2014; Morrell *et al*., 2014). However, the patterns of geographical distribution of the functional *btr1* and *btr2* mutations challenged the simplicity of the east–west model – in addition to differentiation of the *btr1* and *btr2* mutations along the east–west gradient in the Fertile Crescent, another latitudinal cline in the *btr1* and *btr2* allele frequencies became apparent (Pourkheirandish *et al*., 2015).

Our findings based on the origin of the domestication sweep loci expand the east–west model by suggesting that not only two but multiple protodomesticated lineages may have existed in the past in the Levantine and Zagros clusters of the origin of agriculture. The presence of a third mutation conferring the nonbrittle rachis phenotype of domesticated barley supports this hypothesis (Civáň & Brown, 2017).

Recently, based on the genome‐wide SNP genotyping data, Poets *et al*. (2015) presented the mosaic model of barley domestication, which suggests that the genomes of modern barley landraces consist of a mixture of ancestral blocks originating in the five wild barley populations from different parts of the Fertile Crescent (Allaby, 2015; Poets *et al*., 2015). We found that, in contrast to the domestication sweep regions, the neutral partition of the domesticated barley genomes comprised ancestral blocks that descended from all nine wild barley populations corroborating the mosaic model. Moreover, the ancestral patterns at the neutral loci were not very similar across the genotypes. This indicates that, after domestication, the gene flow between the wild and domesticated subspecies and the domesticates themselves continued reshuffling the ancestral blocks in the modern domesticated genotypes, thus erasing the genome‐wide signatures of independent protodomesticated lineages. A simulation study demonstrated that, in the case of neutral markers, the gene flow between the independent domestication lineages indeed hinders identification of the founder events (Allaby *et al*., 2008). By contrast, the domestication sweep loci had nearly uniform ancestry patterns across the genotypes. This shows the importance of retaining specific ancestral alleles of the domestication genes, which are critical for maintaining the domestication syndrome traits.

Involvement of gene flow in domestication has been documented in other crop species (Huang *et al*., 2012; Civáň *et al*., 2013; Hufford *et al*., 2013). The model of rice domestication is arguably the most vivid example. In rice, two different, possibly extinct wild *Oryza rufipogon* populations were ancestors of the *indica* and *japonica* domesticated subspecies; however, the domestication sweep loci originated once in *japonica* and were later introgressed into the *indica* lineage (Fuller, 2011; Huang *et al*., 2012; Huang & Han, 2015; Choi *et al*., 2017; but see Civáň *et al*., 2015). We suggest that, in contrast to the rice model, in the barley domestication history, gene flow was not an isolated event but probably a continuous process, which ensued in the early domestication era and was apparently facilitated by modern breeding. Indeed, the genome of a 6000‐yr‐old barley landrace carried signatures of the wild barley introgressions, thus confirming instances of the gene flow in the early domesticates (Mascher *et al*., 2016).

We have yet to understand the exact nature and sequence of demographic events that formed the complex mosaic ancestry patterns during the apparently protracted process of barley domestication. Involvement of several wild populations and abundant continuous gene flow in the process of barley domestication greatly complicates explicit modeling of a realistic demographic history of its domestication. What is clear, however, is that it was not constrained to a single center of domestication and involved intensive exchange of the early domesticates between the Neolithic farming communities. Recent evidence predicting migration between the early agriculturalist settlements of the eastern and western parts of the Fertile Crescent hints at the likelihood of such a scenario (Lazaridis *et al*., 2016).

To further unravel barley domestication history, characterization of direct targets of selection within the domestication sweep regions is of the utmost importance. Our catalog of the candidate domestication loci will facilitate future efforts to characterize novel domestication genes, which modulate as yet unstudied aspects of the barley domestication syndrome.

## Author contributions

A.P. and M.v.K. planned and designed the research. J.A. and C.B conducted the enrichment sequencing. A.P. analyzed the data. A.P. and M.v.K. wrote and revised the manuscript.

## Supporting information

Please note: Wiley Blackwell are not responsible for the content or functionality of any Supporting Information supplied by the authors. Any queries (other than missing material) should be directed to the *New Phytologist* Central Office.

**Fig. S1** Selection of target genes.
**Fig. S2** The data analysis pipeline – read filtering, mapping, SNP calling and genotyping.
**Fig. S3** Characteristics of coverage.
**Fig. S4** Distribution of SNP markers over the barley chromosomes.
**Fig. S5** Correlation of the ancestry coefficients estimated using faststructure and instruct.
**Fig. S6** Genomic signatures of domestication selective sweeps (Mascher_2013 map).
**Fig. S7** Procedure for estimating the ancestry of domesticated barley haplotypes.
**Fig. S8** Population structure of wild barley (K = 9) determined by faststructure and instruct.
**Fig. S9** The maximum likelihood (ML) unrooted phylogeny of 230 nonadmixed barley accessions.
**Fig. S10** The Neighbor‐Net phylogenetic network of 359 barley accessions.
**Fig. S11** Distribution of the wild barley populations within the Fertile Crescent.
**Fig. S12** Distribution of the wild barley populations in Israel, the West Bank and Gaza.
**Fig. S13** Unsorted ancestral palettes of the candidate domestication loci.
**Fig. S14** Sorted ancestry palettes of the domesticated barley genotypes.
**Fig. S15** Estimation of the median ancestry coefficients in the unbalanced subgroups of loci.
**Fig. S16** Heatmaps of the pairwise ancestry similarity coefficients.
**Fig. S17** A simplified candidate demographic model implying multiple domestication lineages.
**Fig. S18** A simplified candidate demographic model implying a single domestication lineage.
**Table S1** Characteristics of wild and cultivated barley accessions
**Table S2** Sequences selected for targeted enrichment design
**Table S3** Gene ontology (GO) terms of selected Arabidopsis genes
**Table S4** Composition of the mapping reference
**Table S5** Characteristics of the enrichment assay and SNP calling
**Table S6** Characteristics of outlier loci identified by the selection scans
**Table S7** Statistical assessment of wild barley population clustering by ‘smartpca’
**Methods S1** Selection of genes for targeted enrichment assay.
**Methods S2** Mapping reference design.
**Methods S3** Quality check, mapping and SNP calling pipeline.
**Notes S1** Characteristics of the enrichment assay.
**Notes S2** Wild barley population structure – a note of caution.
