# Supplementary material for: Targeted resequencing reveals genomic signatures of barley domestication
Source: New Phytol. 2018 Mar 12;218(3):1247–59. doi: 10.1111/nph.15077 (PMC5947139; doi:10.1111/nph.15077)
Supplement: Supplementary file 1 — Fig. S1 Selection of target genes. Fig. S2 The data analysis pipeline – read filtering, mapping, SNP calling and genotyping. Fig. S3 Characteristics of coverage. Fig. S4 Distribution of SNP markers over the barley chromosomes. Fig. S5 Correlation of the ancestry coefficients estimated using faststructure and instruct. Fig. S6 Genomic signatures of domestication selective sweeps (Mascher_2013 map). Fig. S7 Procedure for estimating the ancestry of domesticated barley haplotypes. Fig. S8 Population structure of wild barley (K = 9) determined by faststructure and instruct. Fig. S9 The maximum likelihood (ML) unrooted phylogeny of 230 nonadmixed barley accessions. Fig. S10 The Neighbor‐Net phylogenetic network of 359 barley accessions. Fig. S11 Distribution of the wild barley populations within the Fertile Crescent. Fig. S12 Distribution of the wild barley populations in Israel, the West Bank and Gaza. Fig. S13 Unsorted ancestral palettes of the candidate domestication loci. Fig. S14 Sorted ancestry palettes of the domesticated barley genotypes. Fig. S15 Estimation of the median ancestry coefficients in the unbalanced subgroups of loci. Fig. S16 Heatmaps of the pairwise ancestry similarity coefficients. Fig. S17 A simplified candidate demographic model implying multiple domestication lineages. Fig. S18 A simplified candidate demographic model implying a single domestication lineage. Table S1 Characteristics of wild and cultivated barley accessions Table S2 Sequences selected for targeted enrichment design Table S3 Gene ontology (GO) terms of selected Arabidopsis genes Table S4 Composition of the mapping reference Table S5 Characteristics of the enrichment assay and SNP calling Table S6 Characteristics of outlier loci identified by the selection scans Table S7 Statistical assessment of wild barley population clustering by ‘smartpca’ Methods S1 Selection of genes for targeted enrichment assay. Methods S2 Mapping reference design. Methods S3 Quality check, mapping and SNP c [file NPH-218-1247-s001.pdf]

***New Phytologist* Supporting Information**

Article title: Targeted resequencing reveals genomic signatures of barley domestication

Authors: Artem Pankin, Janine Altmüller, Christian Becker, Maria von Korff

Article acceptance date: 5 February 2018

Supporting Figures

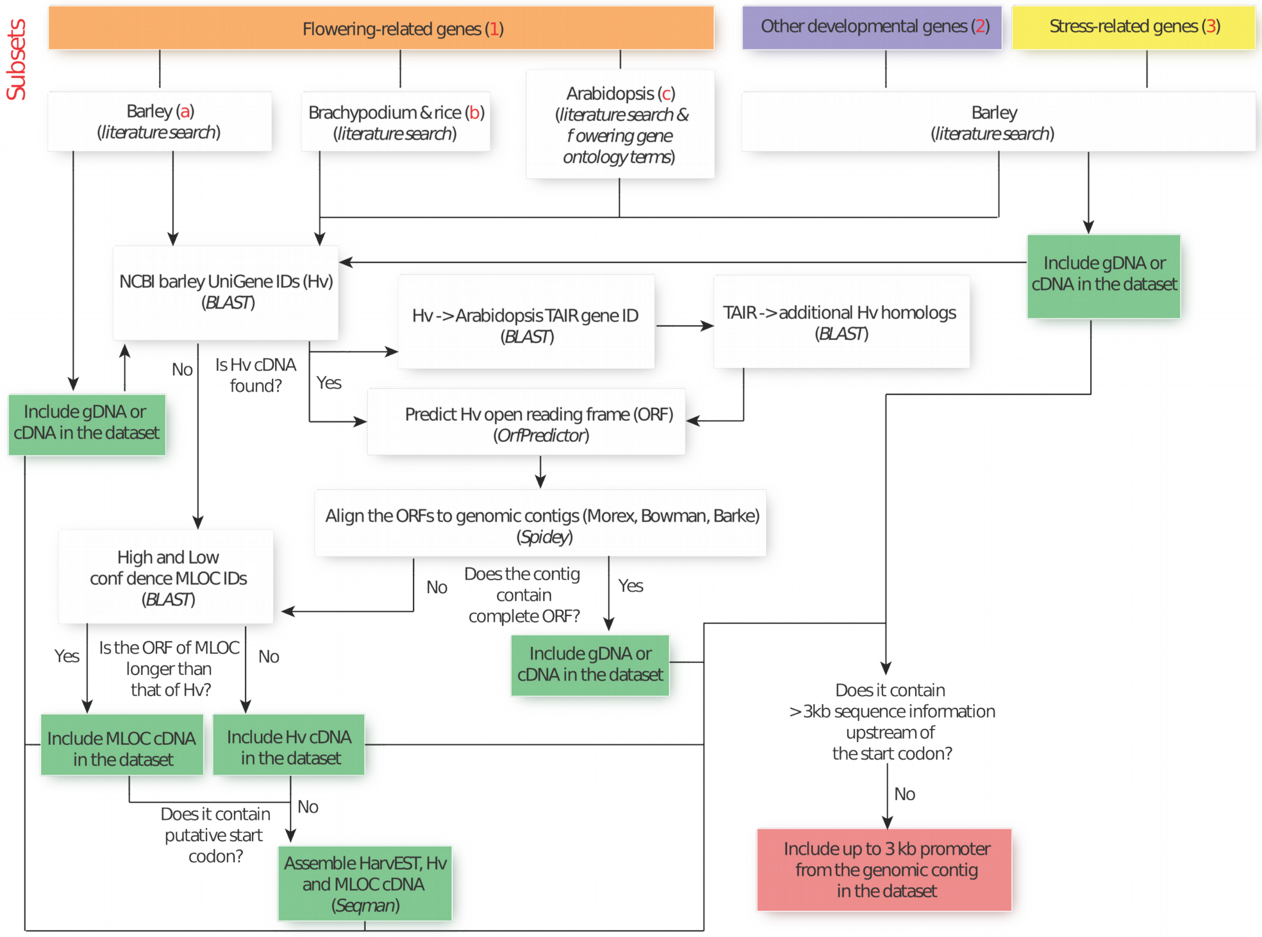

Figure S1 Selection of target genes.

Subsets of three different functional categories of genes are highlighted in orange, violet and yellow. The output steps of the decision-making processes of selecting gene body sequences and promoter regions are highlighted in green and red, respectively.

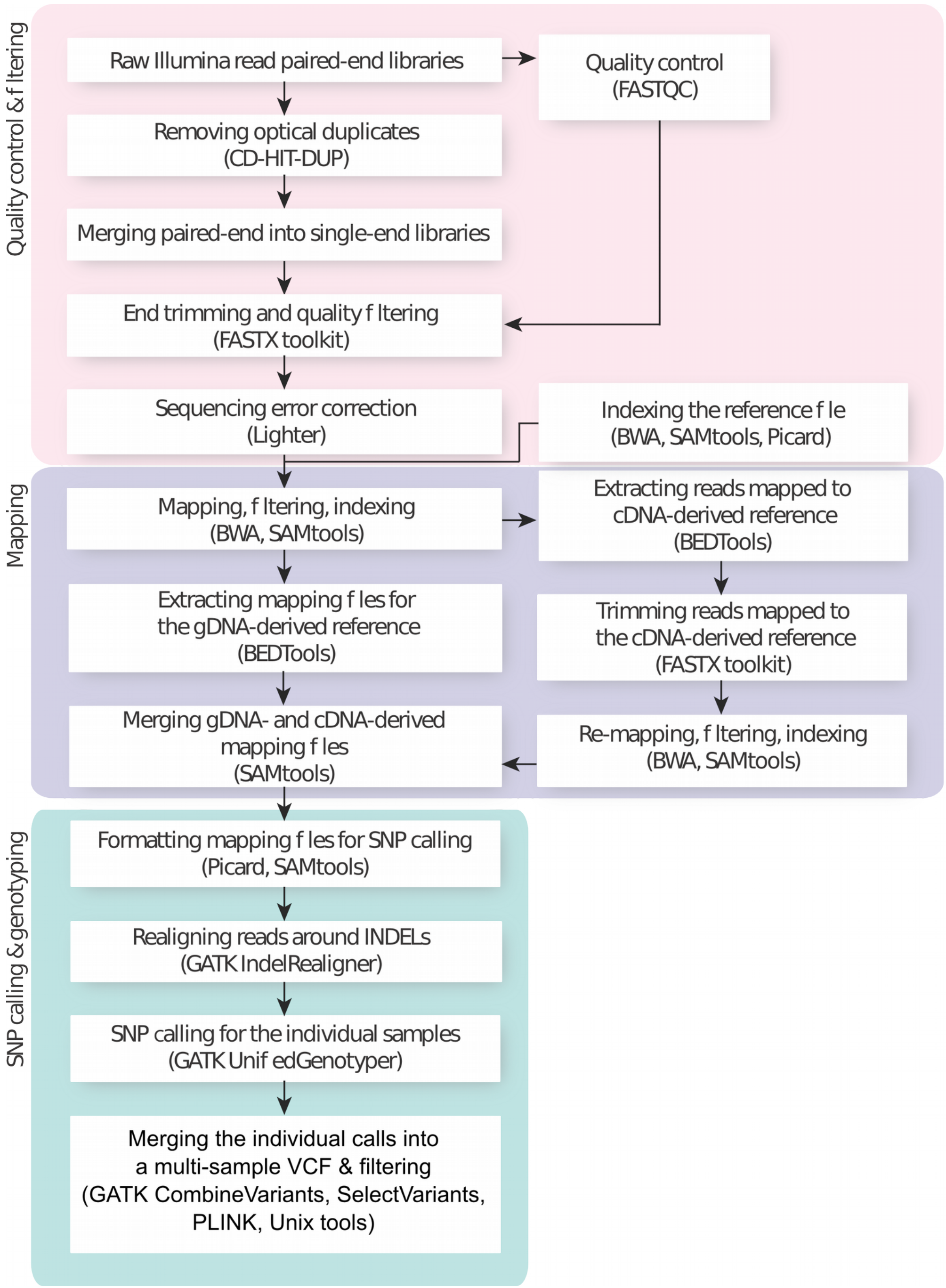

Figure S2 The data analysis pipeline - read filtering, mapping, SNP calling and genotyping.

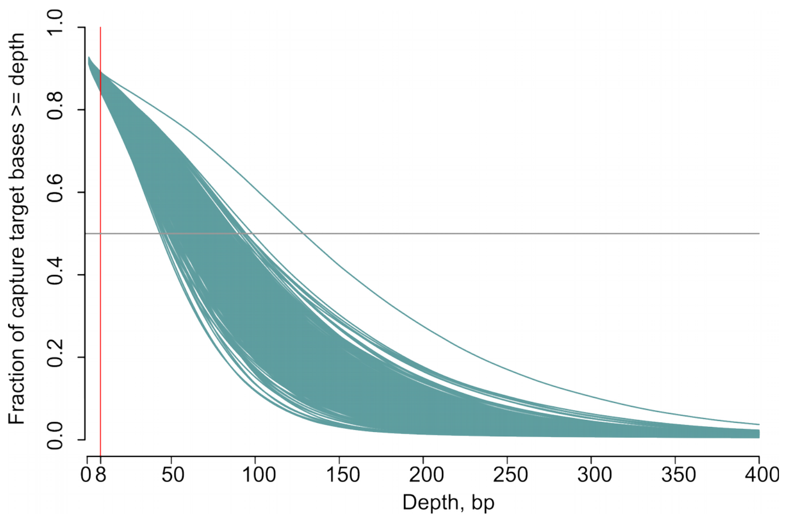

Figure S3 Characteristics of coverage.

A fraction of target nucleotides covered at a certain depth in the individual samples shown as cyan curves. A cut-off coverage threshold for the SNP calling and the median coverage are shown as vertical red and horizontal gray lines, respectively.

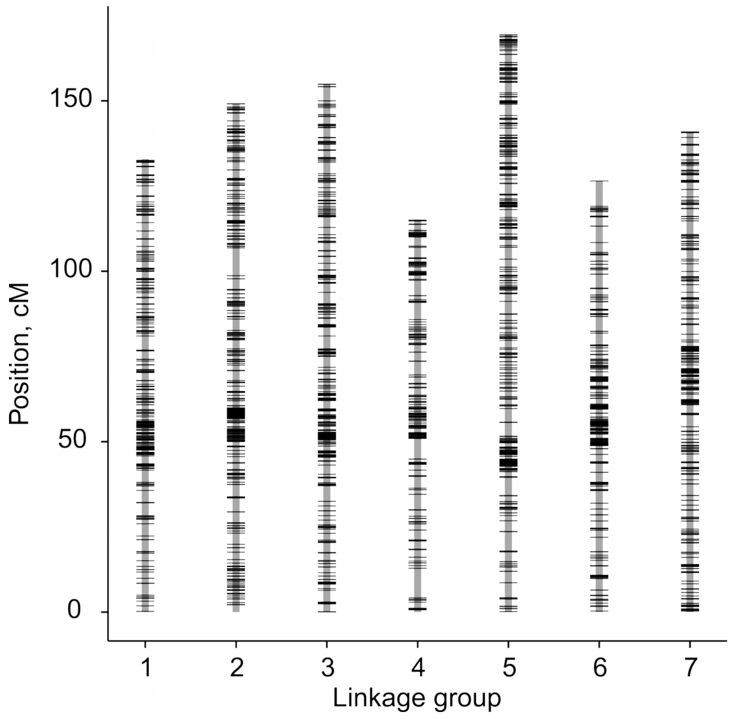

Figure S4 Distribution of SNP markers over the barley chromosomes.

Mapping location of the SNP markers on barley linkage group based on the Mascher_2013 POPSEQ map (Mascher e*t al.*, 2013). The linkage groups and marker positions are shown as vertical gray and horizontal black bars, respectively.

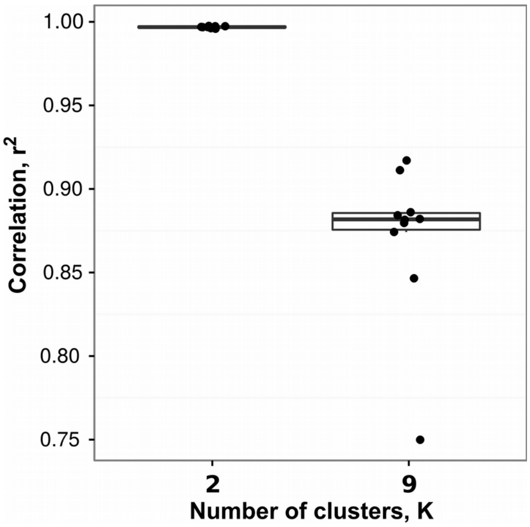

Figure S5 Correlation of the ancestry coefficients estimated using fastSTRUCTURE and INSTRUCT models for the number of clusters K=2 (wild and domesticated) and K=9 (wild).

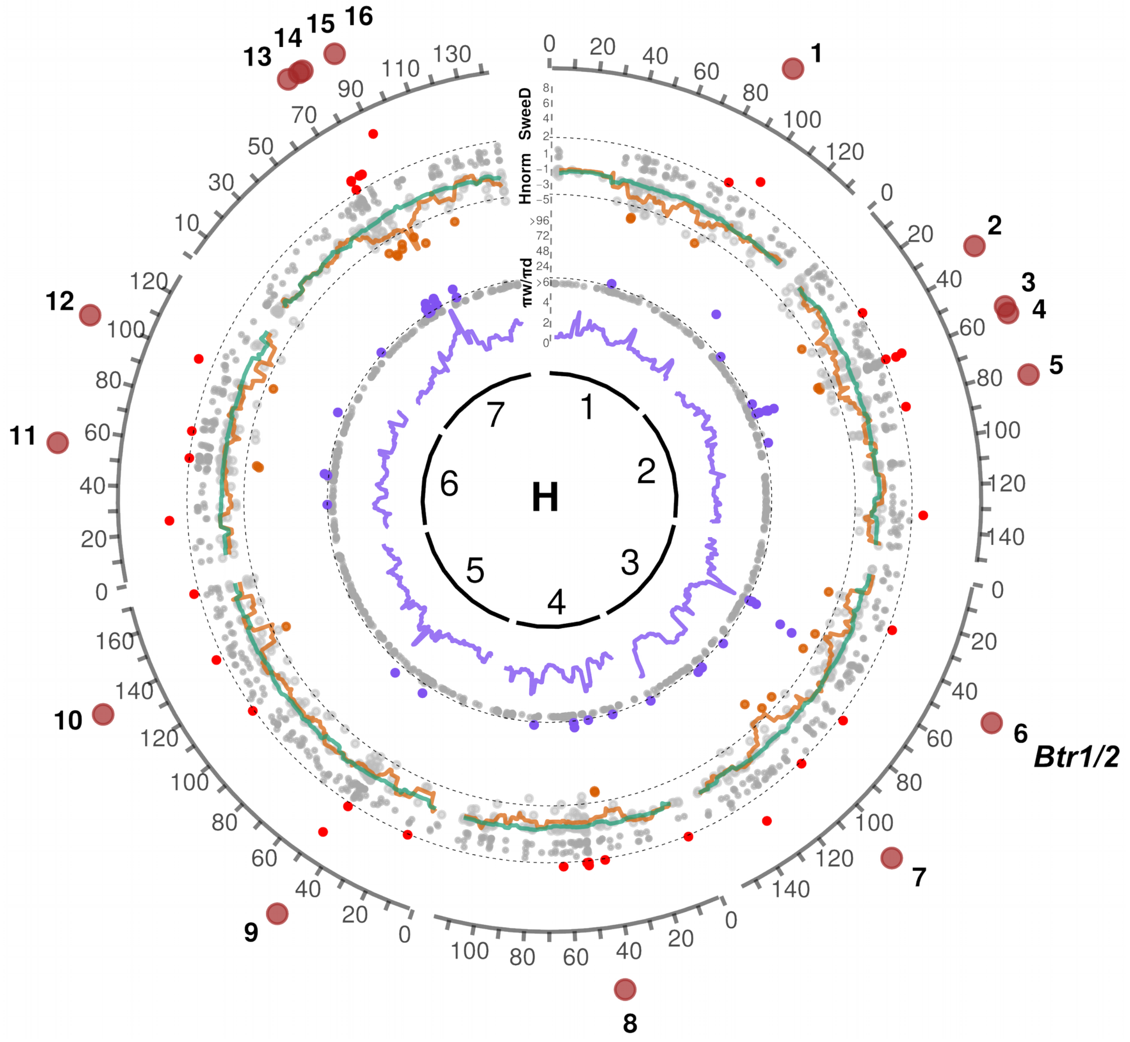

Figure S6 Genomic signatures of domestication selective sweeps (Mascher_2013 map). Genome scans for signatures of selection associated with domestication. The sliding-window and individual-target values are shown as lines and points, respectively. The innermost circle represents barley linkage groups (H) followed by the diversity reduction index (π_{wild}/π_{dom}) (violet); the normalized Fay&Wu's H_{norm} statistics for the wild (green) and domesticated (orange) groups; and the composite likelihood ratio statistics (SweeD CLR) for the domesticated group (red). The outlier thresholds are shown by dashed lines and the non-outlier loci are shown as gray dots for all the tests. 16 candidate selected regions supported by at least two of the statistics are shown as brown circles on the outermost layer. *Btr1/2* – brittle rachis domestication genes (Pourkheirandish *et al.*, 2015).

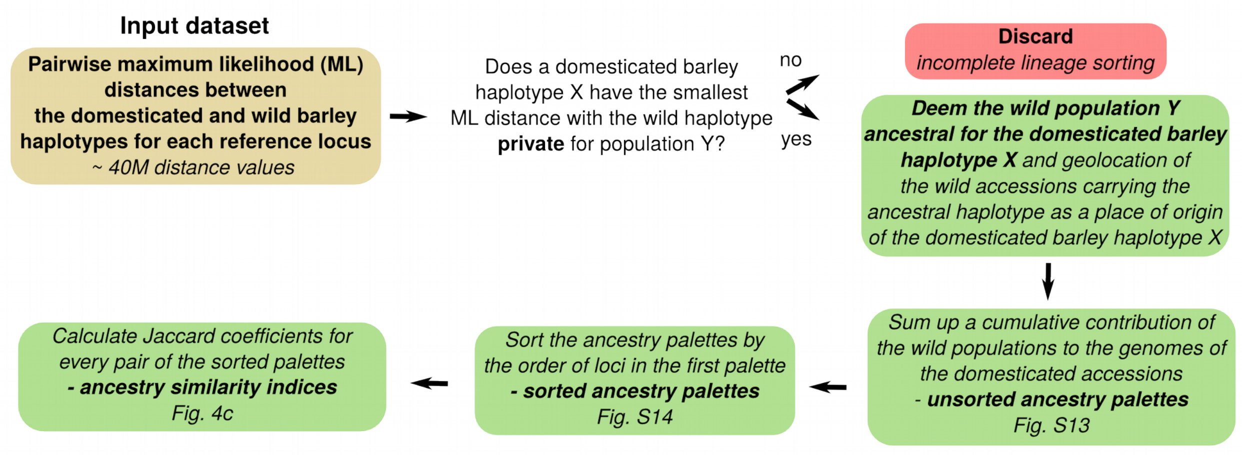

Figure S7 Procedure for estimating the ancestry of domesticated barley haplotypes.

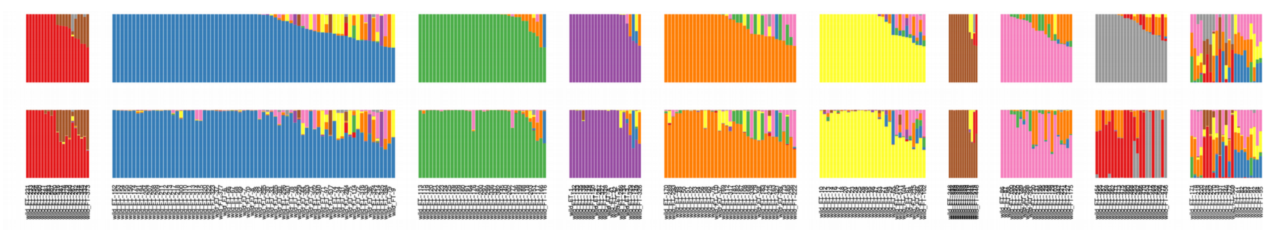

Figure S8 Population structure of wild barley (K=9) determined by fastSTRUCTURE and INSTRUCT models – upper and lower panels, respectively. Vertical bars correspond to individual genotypes and colors indicate their membership in the nine subpopulations.

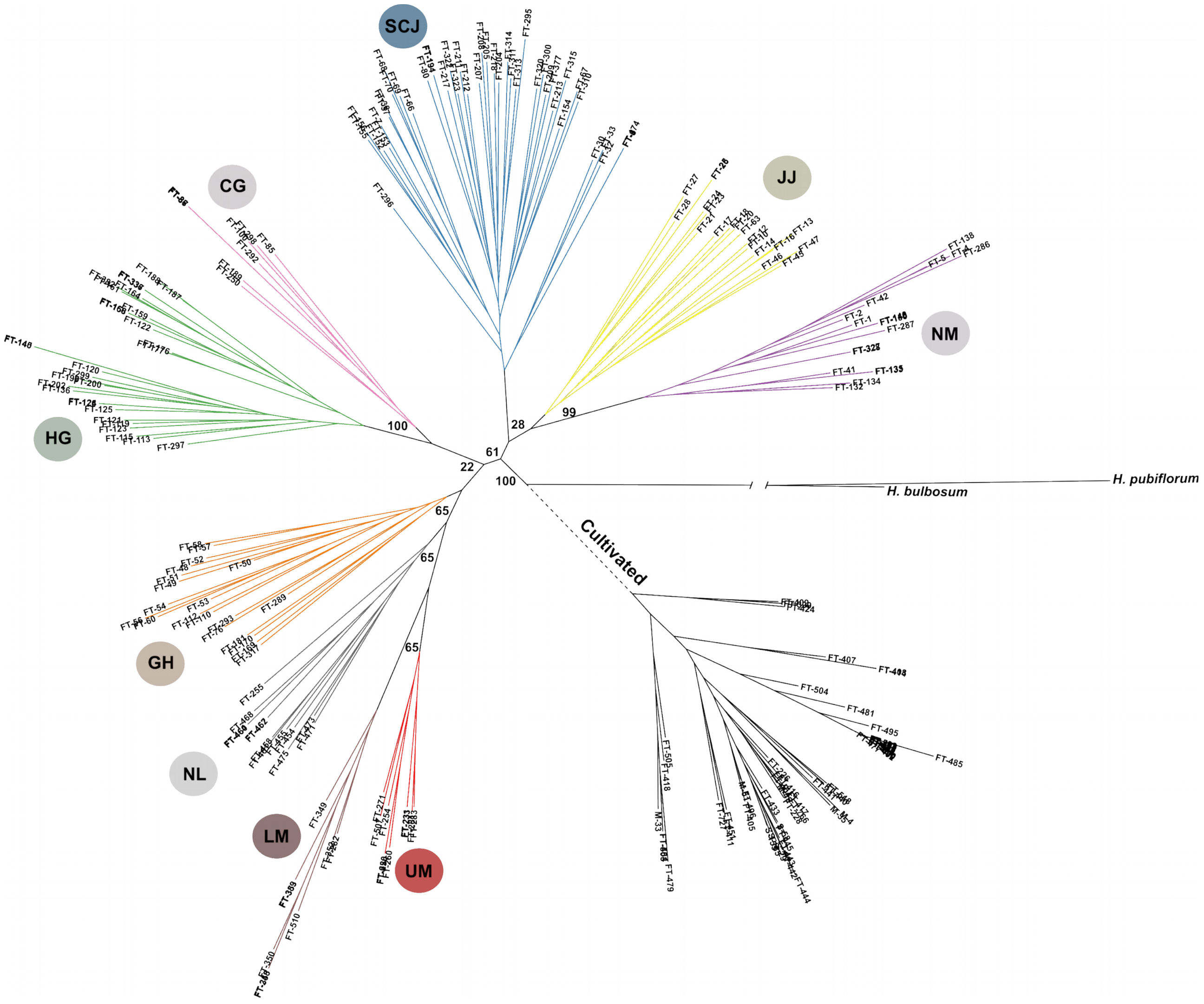

Figure S9 The Maximum Likelihood (ML) unrooted phylogeny of 230 non-admixed barley accessions.

Colored clusters correspond to the nine wild barley (*H. vulgare* ssp. *spontaneum*) populations (membership coefficient > 95% in a single population). Carmel & Galilee (CG; pink); Golan Heights (GH; orange); Hula Valley & Galilee (HG; green); Judean Desert & Jordan Valley (JJ; yellow); Lower Mesopotamia (LM; brown); Negev Mountains (NM; magenta); North Levant (NL; grey); Sharon, Coastal Plain & Judean Lowlands (SCJ; blue); Upper Mesopotamia (UM; red). Cultivated barley (*H. vulgare* ssp. *vulgare*) is shown as a black cluster. The dashed line indicates that the phylogenetic placement of the cultivated barley cluster may be uncertain due to its complex hybrid origin. Wild barley *H. bulbosum* and *H. pubiflorum* are used as distant outgroup species and the length of the outgroup branch is artificially shortened. The bootstrap values are shown at the corresponding nodes.

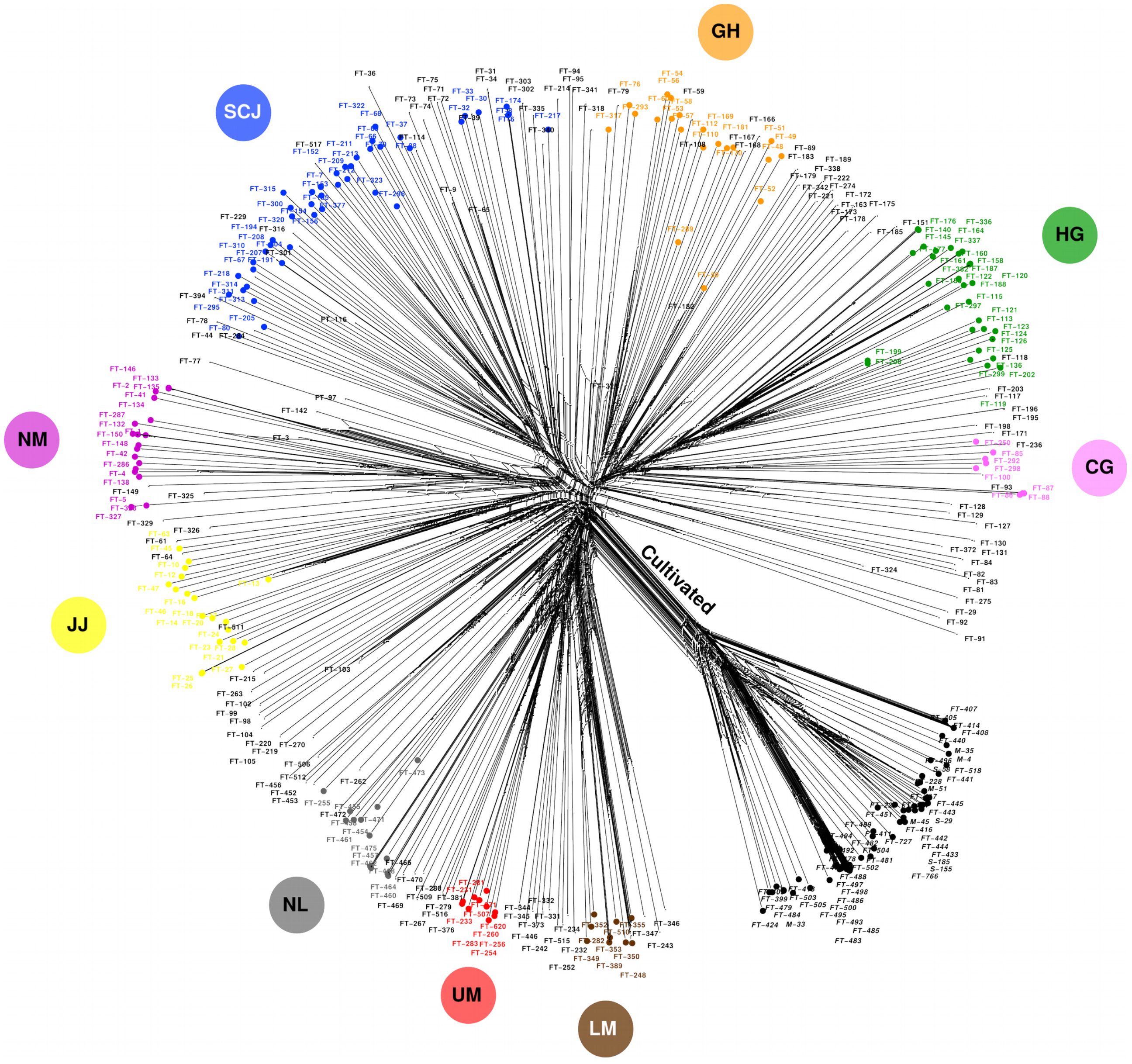

Figure S10 The Neighbor-Net phylogenetic network of 359 barley accessions.

Colored nodes correspond to the wild barley (*H. vulgare* ssp. *spontaneum*) genotypes assigned to any of the nine populations (membership coefficient > 95%). The wild barley genotypes with the membership < 95% are shown by node labels in a regular black font. The population label abbreviations are as in **Fig. S9**. Cultivated barley (*H. vulgare* ssp. *vulgare*) genotypes are shown by black nodes and labels in bold.

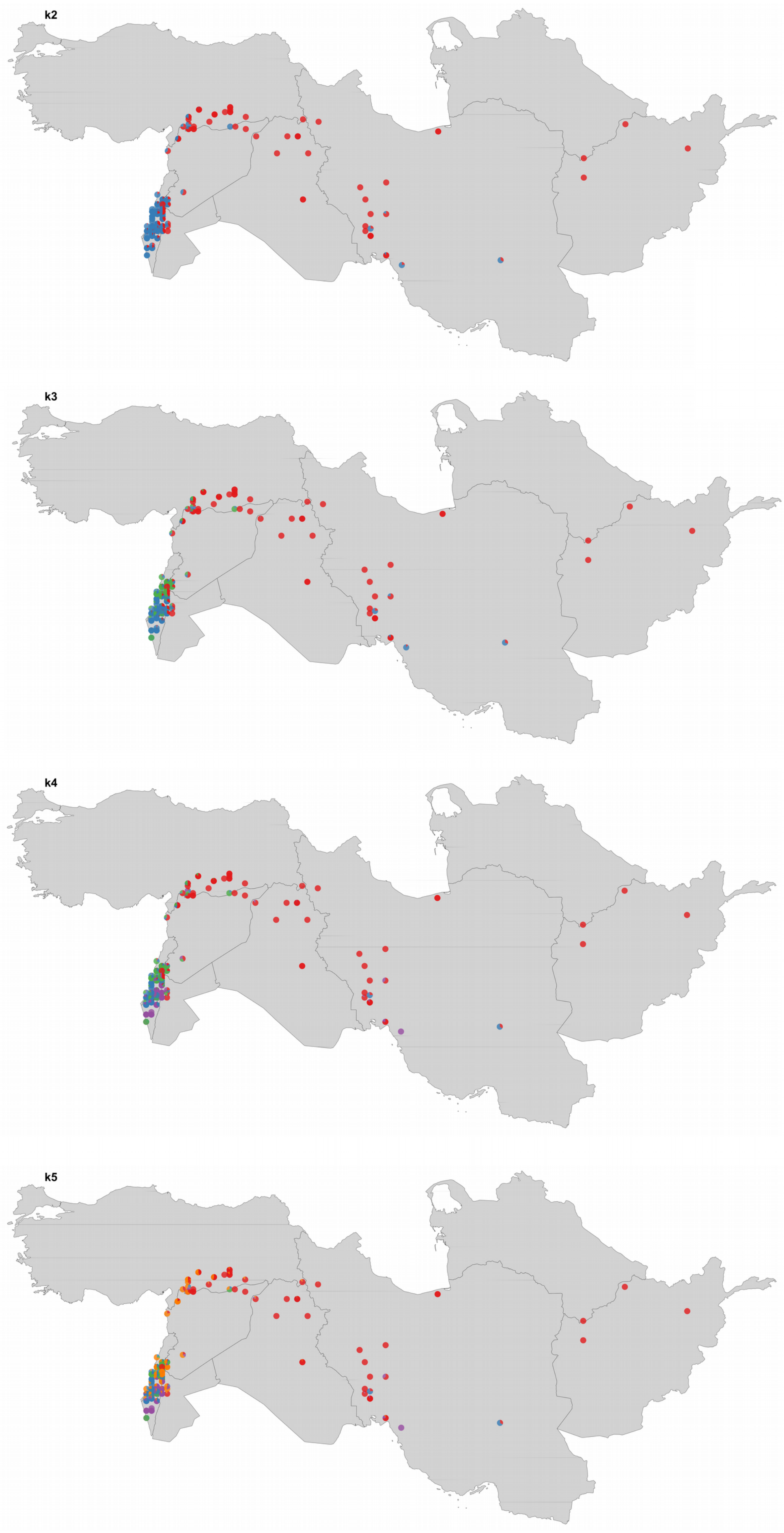

Figure S11 Distribution of the wild barley populations within the Fertile Crescent. The pie charts, reflecting ancestral composition of the individual genotypes as determined by fastSTRUCTURE for K from 2 to 9, are shown at the geographic location of the genotypes. See **Fig. S9** for the color legend.

Figure S11 *(continued)* Distribution of the wild barley populations within the Fertile Crescent. The pie charts, reflecting ancestral composition of the individual genotypes as determined by fastSTRUCTURE for K from 2 to 9, are shown at the geographic locations of the genotypes. See **Fig. S9** for the color legend.

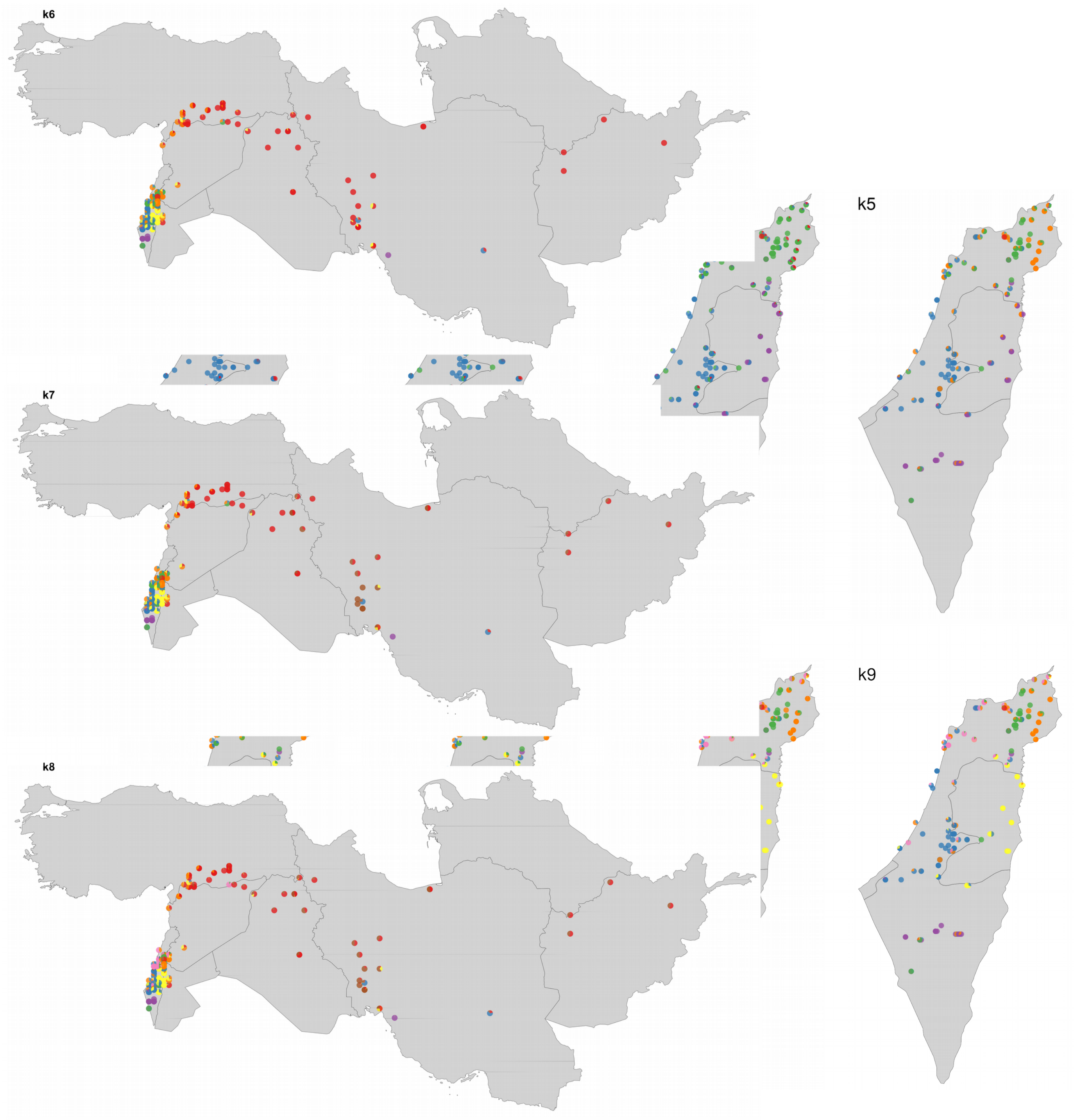

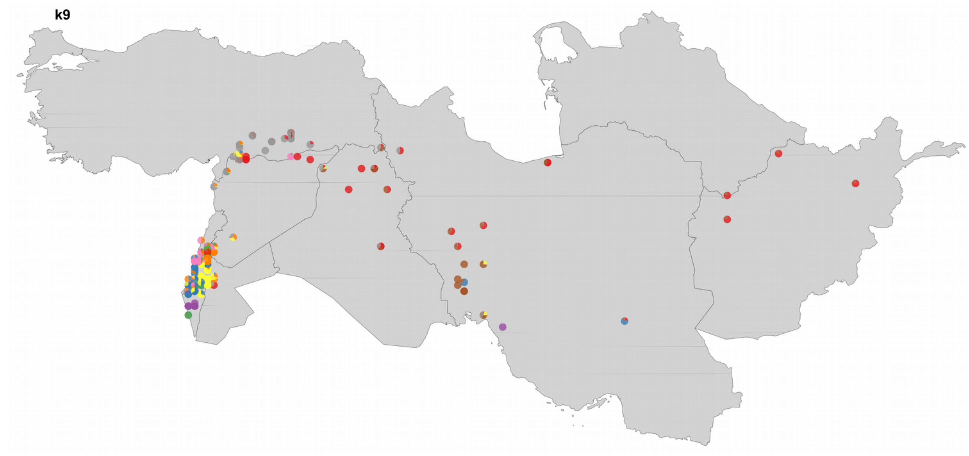

Figure S12 Distribution of the wild barley populations within Israel, the West Bank and Gaza.

The pie charts, reflecting ancestral

composition of the individual genotypes as determined by fastSTRUCTURE for K from 2 to 9, are connected to their geographic location. See **Fig. S9** for the color legend.

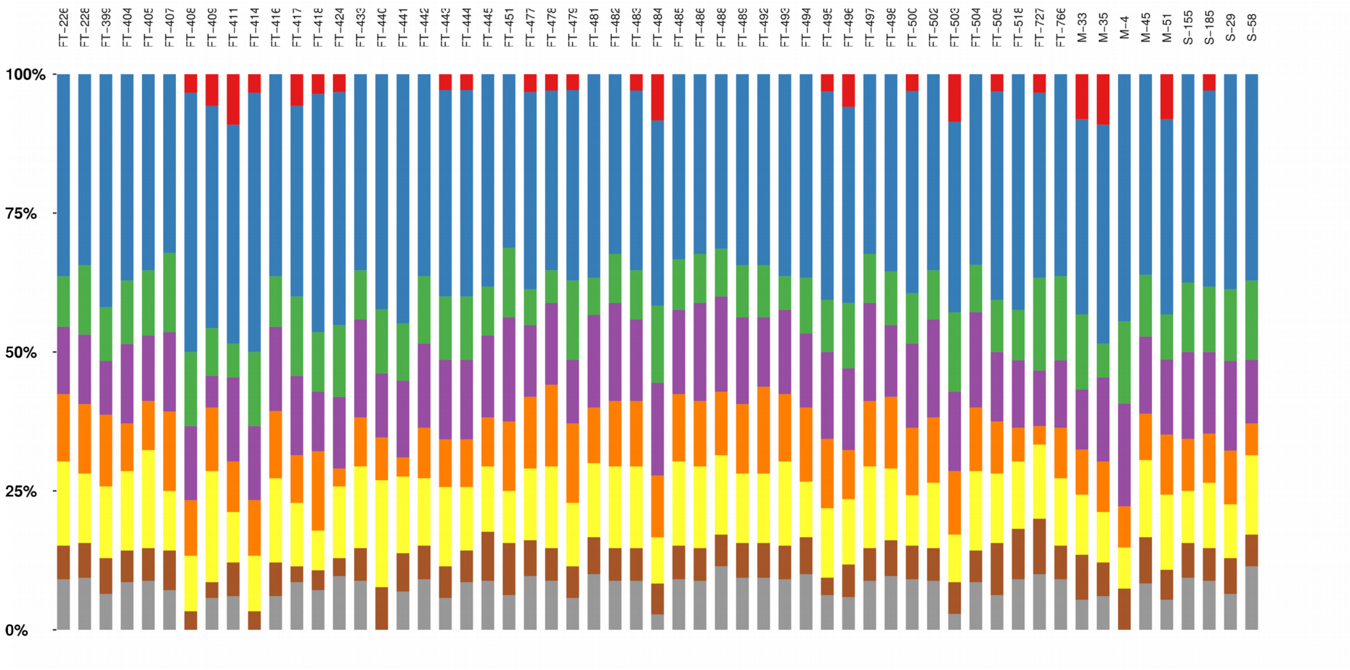

Figure S13 Unsorted ancestral palettes of the candidate domestication loci demonstrating cumulative contribution of the wild populations to the ancestry of the domesticated genomes.

See **Fig. S9** for the color legend.

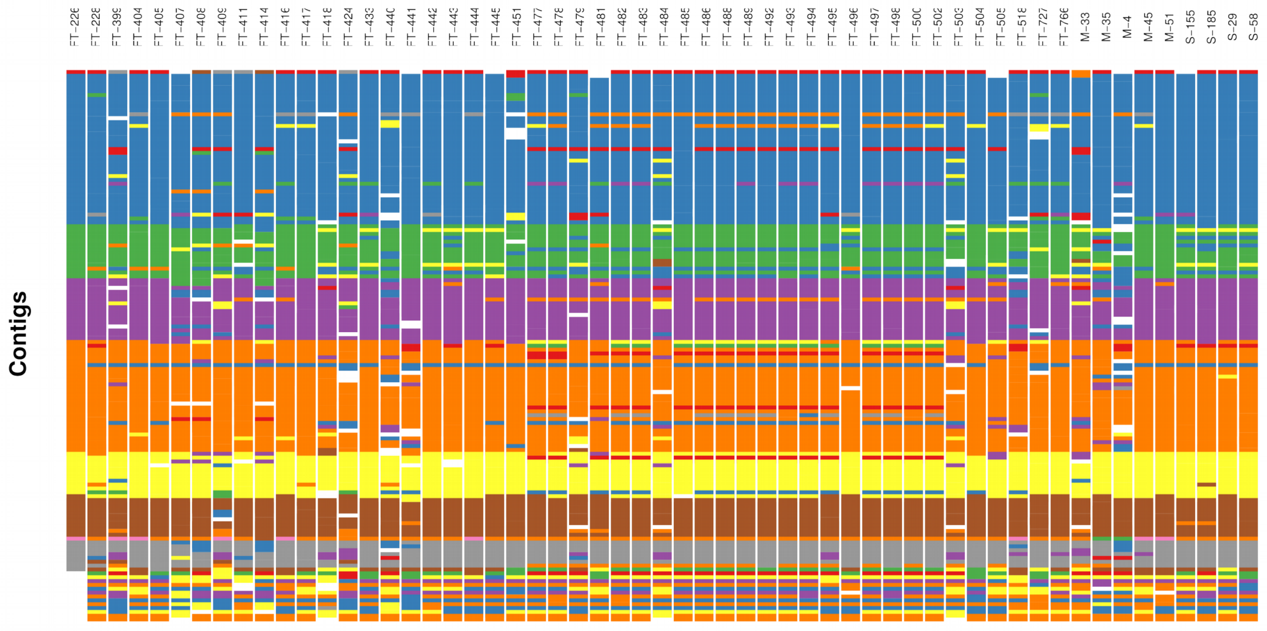

Figure S14 Illustration of the sorted ancestry palettes of the domesticated barley genotypes (neutral and domesticated sweep loci with ancestry assigned in > 80% of the genotypes).

The palettes were sorted according to the order of the contigs in the FT226 ancestry palette. See **Fig. S9** for the color legend. Missing values are shown as blanks.

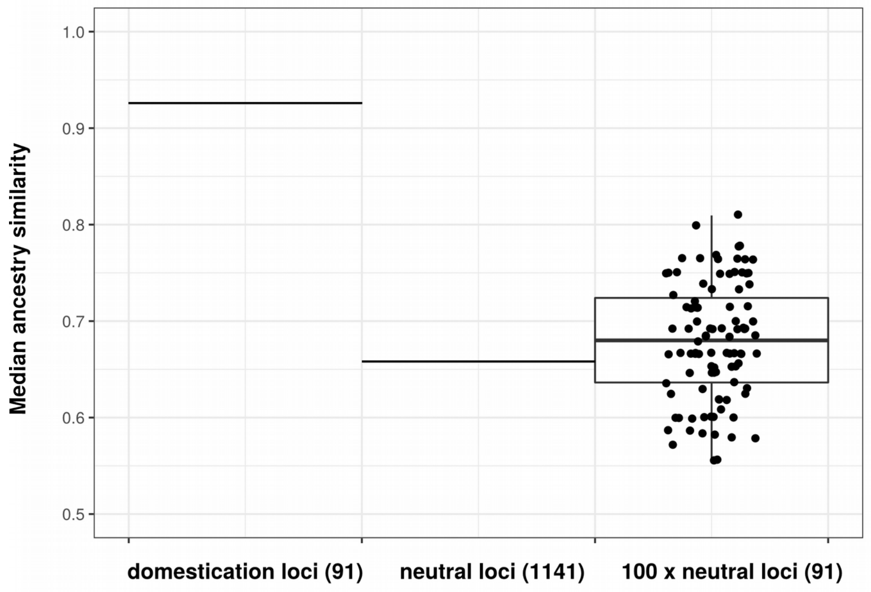

Figure S15 Estimation of the median ancestry coefficients in the unbalanced subgroups of loci (91 domestication and 1141 neutral loci) and in the 100 randomly drawn subsets of 91 neutral loci.

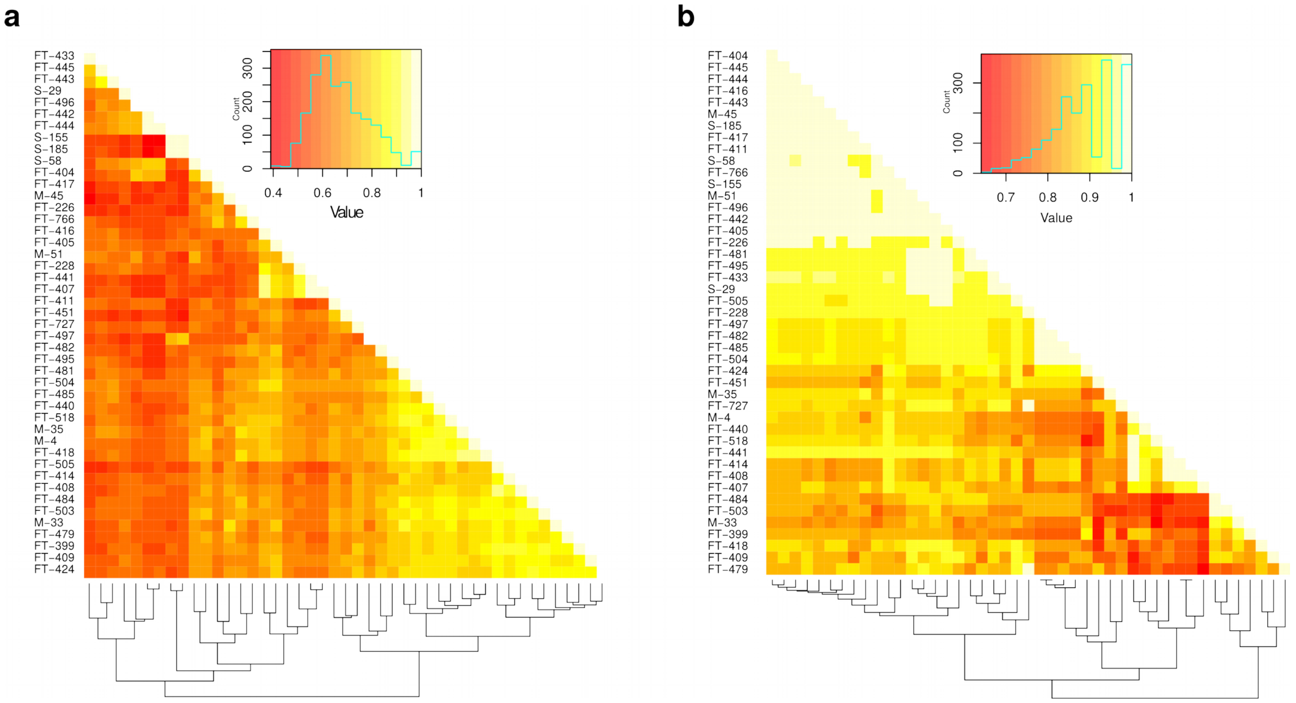

Figure S16 Heatmaps of the pairwise ancestry similarity coefficients.

The insets represent the color legend and contain the histograms of the ancestry similarity coefficients calculated for the neutral (**a**) and domestication sweep loci (**b**).

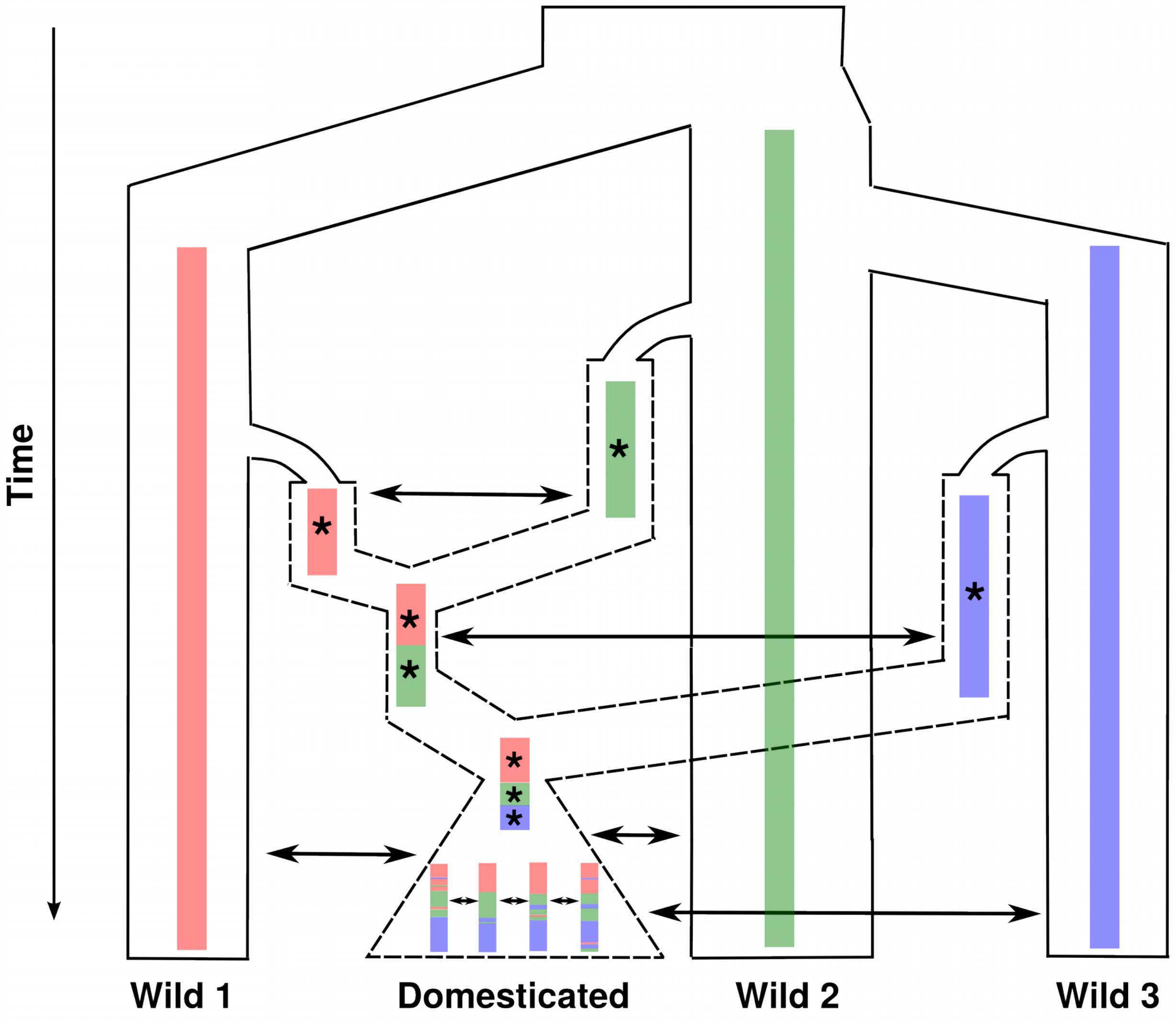

Figure S17 A simplified candidate demographic model implying multiple domestication lineages proposed based on the ancestry patterns of the domesticated barley genomes.

Red, green and blue colors represent three founder populations of wild barley (solid lines) and corresponding independent domestication lineages (dashed lines). The colored bars are analogous to the ancestry palettes. The double-sided arrows illustrate gene flow between the lineages. Occurrences of the selective sweeps are shown as asterisks.

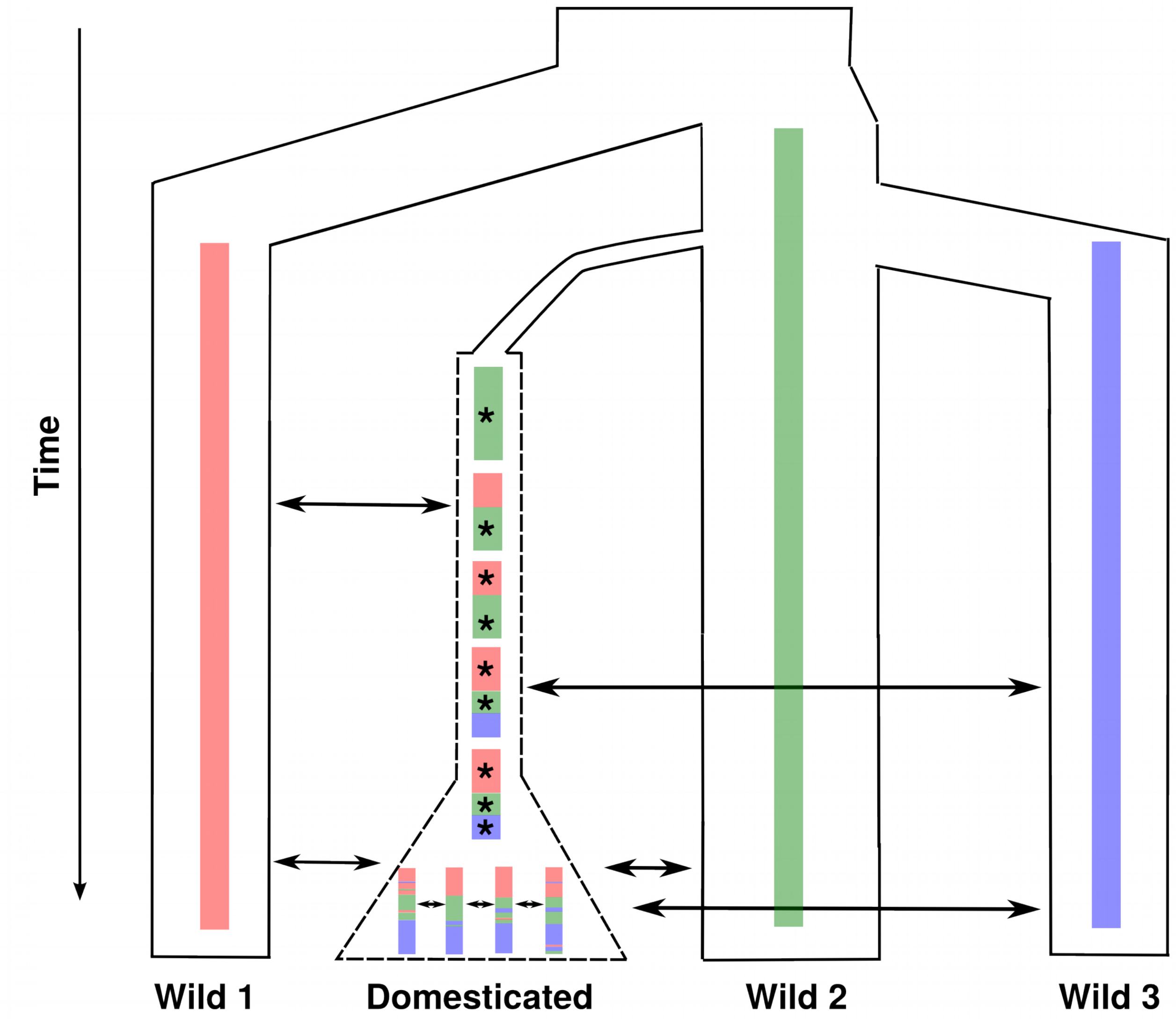

Figure S18 A simplified candidate demographic model implying a single domestication lineage proposed based on the ancestry patterns of the domesticated barley genomes.

Red, green and blue colors represent three founder populations of wild barley (solid lines) and corresponding domestication lineage (dashed lines). The colored bars are analogous to the ancestry palettes. The double-sided arrows illustrate gene flow between the lineages. Occurrences of the selective sweeps are shown as asterisks.

Supporting Methods.

Methods S1 Selection of genes for targeted enrichment assay

A set of genic and upstream regulatory sequences selected for enrichment comprised a comprehensive subset of loci related to flowering time and development of meristem and inflorescences. Additionally, a set genes related to agronomic traits putatively affected by domestication, e.g. tillering, seed dormancy, carbohydrate metabolism, was selected. First, scientific literature was mined for the genes implicated in the aforementioned processes and the corresponding nucleotide sequences were extracted from NCBI GenBank. Second, flowering genes from the other grass species, such as Brachypodium and rice, were selected (Higgins et al., 2010). Third, a set of 259 Arabidopsis genes characterized by the flowering-related gene ontology (GO) terms that have been confirmed experimentally was assembled (**Table S3**). The barley homologs of all these genes were extracted from the NCBI barley UniGene set (Hv cDNA, cv. Haruna Nijo, build 59) either by the BLASTN search (e-value < 1e-7) or, in the case of Arabidopsis genes, by searching the annotation table downloaded from the NCBI UniGene server (ftp://ftp.ncbi.nih.gov/repository/UniGene/Hordeum_vulgare). This table was further used to reciprocally extract additional Hv homologs based on the Arabidopsis gene identifiers. If the BLAST search failed to identify a reliable Hv homolog, the homologs were searched in the barley High and Low confidence genes (MLOC cDNA) (IBGSC, 2012) and in the HarvEST unigene assembly 35 (http://harvest.ucr.edu).

Open reading frames (ORF) of Hv cDNA were predicted using OrfPredictor guided by the BLASTX search against Arabidopsis TAIR 10 database (Min *et al.*, 2005). The predicted ORFs were aligned to the genomic contigs of barley cultivars Morex, Bowman and Barke using the Spidey algorithm implemented in the NCBI toolkit. The ORFs of the selected sequences were categorized as complete or partial based on the presence or absence of putative start and stop codons. The complete complementary DNA (cDNA) were selected and, if the complete cDNA was absent, partial gDNA and cDNA were included in the dataset. For several genes with previously characterized intronic regions, e.g. predicted to contain regulatory elements, complete genomic DNA (gDNA) were selected. In case when only partial cDNA was available, chimeric sequences were assembled from the Hv, MLOC and HarvEST cDNA using SeqMan software (DNASTAR Lasergene®8 Core Suite, Madison, WI, USA). The selected sequences were cross-annotated with NCBI UniGene Hv and IBGSC MLOC identifiers using reciprocal BLASTN (e-value < 1e-05). In addition to the coding regions and introns, the selection contained sequences up to 3 kilobase pairs (Kbp) upstream of the predicted start codons, which presumably corresponded to regulatory promoter regions.

A set of 1000 additional HarvEST genes was randomly selected such that they had no homology to target genes as determined by BLASTN and were evenly spread over all barley linkage groups according to the GenomeZipper map (Mayer *et al.*, 2011). The 100-bp stretches of each of these genes were included in the enrichment library.

The target sequences were filtered and tiled with 100-bp selection baits using Nimblegen proprietary algorithm and the library of baits was synthesized as a part of the SeqCap EZ enrichment kit (design name 130830_BARLEY_MVK_EZ_HX3; Roche NimbleGene, Madison, WI). Barcoded Illumina libraries were individually prepared, then enriched and sequenced in 24-sample pools at the Cologne Center for Genomics facilities following the standard protocols.

Methods S2 Mapping reference design

The genic sequences from a variety of barley genotypes were used to design the enrichment library to ensure that the longest ORF and promoter regions were selected. However, most advanced physical and genetics maps have been developed for the barley cultivar Morex. Since mapping information is essential for the downstream analyses, the so-called Morex genomic contigs were used as a mapping reference provided that they comprised the entire regions tiled by the baits (**Table S7**) (IBGSC, 2012). If such contigs were not available, the genomic contigs of the barley genotypes Bowman and Barke or the templates that were used for the bait design were included in the mapping reference.

To identify the off-target enrichment regions, the Illumina reads from 10 randomly selected barley genotypes were mapped to the complete Morex genome reference set (IBGSC, 2012). All genomic contigs that had at least one read mapped to them were included in the mapping reference. The Morex contigs were masked with “N”s at the regions of > 100 bp that exhibited > 97% homology with the original capture targets. Altogether, the reference genome for mapping comprised 23,408 contigs.

Methods S3 Quality check, mapping and SNP calling pipeline

The quality parameters of the paired-end Illumina libraries were assessed using FastQC tool (v. 0.11.2; http://www.bioinformatics.babraham.ac.uk/projects/fastqc). After filtering out optical duplicates, resulting from a PCR amplification, using the CD-HIT-DUP software (v. 0.5) (Fu *et al.*, 2012), the paired-end read files were merged and henceforth treated as a single-end dataset. Next, based on the FastQC results, the reads were trimmed from both ends to remove low quality sequencing data, filtered to remove the remaining adaptor sequences and low-complexity artifacts

using the FASTX toolkit (v. 0.0.14; http://hannonlab.cshl.edu/fastx_toolkit). The sequencing errors in the dataset were corrected using the Bloom-filter tool Lighter with the conservative set of parameters: k-mer size 23, alpha 0.2, and maximum corrections per read 2 (Song *et al.*, 2014). The reference file was indexed for the downstream processing using Burrows-Wheeler Aligner 0.5.9-r16 (BWA), SAMtools and Picard tools (http://broadinstitute.github.io/picard) (Li and Durbin, 2010; McKenna *et al.*, 2010). The groomed read datasets were mapped onto the reference genome using BWA (modules 'aln' and 'samse') with the following stringency parameters: missing probability (-n) 0.05, maximum number of gaps (-o) 2, and gap extensions (-e) 12. Some of the reference loci were present in the form of cDNA and the gDNA-derived reads mapped onto such targets may generate false positive SNP calls at the intron-exon junctions. To alleviate this problem, the reads that mapped to cDNA-derived targets were extracted, additionally trimmed by 14 bp from each end and remapped following the described procedure. Reads that mapped to several locations were filtered out.

Calling variant (SNP) and invariant sites was performed for each sample library separately using the GATK UnifiedGenotyper walker with the default parameters except for the following flags: -pcr_error_rate 5.0E-02; -output_mode EMIT_ALL_CONFIDENT_SITES. The sites passing the following hard filters: biallelic, allele count (AC) 2 or 0, depth of coverage (DP) > 8, mapping quality (MQ) > 20, Fisher strand (FS) < 60, were selected using GATK SelectVariants walker. The individual VCF files were merged into a multi-sample file using the GATK CombineVariants walker. This pipeline was implemented in a series of bash scripts adapted for high-performance parallelized computation.

Supporting Notes

Notes S1 Characteristics of the enrichment assay

Of all the targets, 88% were selected in a form of cDNA and 85% comprised putative promoter regulatory regions > 100 bp. The target sequences were mined from various barley genomic and transcript databases and the predicted open reading frames (ORF) of 126 genes were longer than those of the MLOC genes currently used as a barley reference gene set (IBGSC, 2012). For 52 % of the genes the complete ORFs could be mapped to the IBGSC Morex contigs, whereas the rest of the ORFs were partially or completely absent from the IBGSC reference genome. These apparently represent either the genic regions not yet incorporated in the Morex reference genome or the unique allelic variants.

To attenuate effects of the biased selection of genes on the estimates of genetic diversity, we selected fragments of 1000 random genes spread over the barley chromosomes. The enrichment

design baits tiled in total 2.42 Mbp of the barley gene space.

It has been shown that the hybridization-based enrichment assays, particularly NimbleGen SeqCap, are prone to generate off-target reads in the human exome capture assays (Bodi et al. 2013). In the human exome sequencing, large high-quality SNP datasets that originate from the off-target enrichment regions have been documented (Guo *et al.* 2012). Likewise, in this study, the size of the off-target captured regions was approximately six times larger than the size of the target capture design and yielded 1.6x more high-quality SNPs than the target regions (**Table S4**; filtered dataset) - a ratio similar to the findings of Guo *et al.* (2012). It is noteworthy that most of the off-target captured regions resided outside of the predicted CDS. This fact strongly suggests that barley genomes contains multiple pseudogenized copies of the genes targeted in our study, which, according to the earlier proposed model, presumably associate with the mobile elements (Wicker *et al.* 2011).

Notes S2 Wild barley population structure – a note of caution

It is noteworthy that the output of the STRUCTURE models is not definitive and frequently a subject of misinterpretations (Kalinowski 2011). The STRUCTURE model does not fit explicit demographic models and assumes a single ancestral group from which all the other populations have radiated. Therefore, various demographic histories may result in identical STRUCTURE patterns (Falush *et al.* 2016). In addition, barley is a predominantly self-pollinating species and therefore the allele frequencies in barley populations significantly deviate from the allele frequencies in the populations following the Hardy-Weinberg principles, which are default assumptions of the STRUCTURE model. Another limitation of the STRUCTURE model is illustrated by our finding that, even at K values > 9, fastSTRUCTURE applied on the combined domesticated and wild barley dataset failed to reveal the mosaic composition of the domesticated barley genomes (“ancient admixture”).

To alleviate these negative effects, we used five different approaches to define wild barley populations – fastSTRUCTURE, INSTRUCT, PCA, ML and Neighbor-Net phylogenies. All the analyses strongly supported structuring genetic variation in wild barley into nine distinct clusters, which apparently represent subpopulations. Nevertheless, we could not rule out that, for example, additional wild barley subpopulations may exist or that some of the genotypes detected as admixed in this study may, in fact, be non-admixed representatives of the under-sampled populations. In future studies, sequencing of additional wild barley genotypes especially from the sparsely sampled regions – the Eastern horn of the Fertile Crescent - may help get further insights into the extent of these issues.

Supporting Tables

Table S5 Characteristics of the enrichment assay and SNP calling.

	**Selected size, Mbp**	**Captured, Mbp**	**Captured CDS, Mbp**	**Homozygous SNPs**		
				Total, with singletons	Total, w/o singletons	Filtered set*
**Target**	2.42	2.24	0.85	121,294	83,752	20,954
**Off-target**	-	11.56	0.48	423,024	270,858	34,682
**Total**	2.42	13.80	1.33	544,318	354,610	55,636

* - minor allele frequency < 0.05; missing data frequency < 0.5

Supporting References

Bodi K, Perera AG, Adams PS, Bintzler D, Dewar K, Grove DS, Kieleczawa J, Lyons RH, Neubert TA, Noll AC, *et al.* 2013. Comparison of commercially available target enrichment methods for next-generation sequencing. *J. Biomol. Tech. JBT* **24**:73–86.

Falush D, Dorp L van, Lawson D. 2016. A tutorial on how (not) to over-interpret STRUCTURE/ADMIXTURE bar plots. *bioRxiv* 66431.

Fu L, Niu B, Zhu Z, Wu S, Li W. 2012. CD-HIT: accelerated for clustering the next-generation sequencing data. *Bioinformatics* **28**:3150–3152.

Guo Y, Long J, He J, Li C-I, Cai Q, Shu X-O, Zheng W, Li C. 2012. Exome sequencing generates high quality data in non-target regions. *BMC Genomics* **13**:1.

Higgins JA, Bailey PC, Laurie DA. 2010. Comparative genomics of flowering time pathways using *Brachypodium distachyon* as a model for the temperate grasses. *PLoS One* **5**:e10065.

International Barley Genome Sequencing Consortium (IBGSC). 2012. A physical, genetic and functional sequence assembly of the barley genome. *Nature* **491**:711–716.

Kalinowski ST. 2011. The computer program STRUCTURE does not reliably identify the main genetic clusters within species: simulations and implications for human population structure. *Heredity* **106**: 625-632.

Li H, Durbin R. 2010. Fast and accurate long-read alignment with Burrows–Wheeler transform. *Bioinformatics* **26**:589-95.

Mascher M, Muehlbauer GJ, Rokhsar DS, Chapman J, Schmutz J, Barry K, Muñoz-Amatriaín M, Close TJ, Wise RP, Schulman AH. 2013. Anchoring and ordering NGS contig assemblies by population sequencing (POPSEQ). *Plant J.* **76**:718–727.

Mayer KF, Martis M, Hedley PE, Šimková H, Liu H, Morris JA, Steuernagel B, Taudien S, Roessner S, Gundlach H. 2011. Unlocking the barley genome by chromosomal and comparative genomics. *Plant Cell* **23**:1249–1263.

McKenna A, Hanna M, Banks E, Sivachenko A, Cibulskis K, Kernytsky A, Garimella K, Altshuler D, Gabriel S, Daly M. 2010. The Genome Analysis Toolkit: a MapReduce framework for analyzing next-generation DNA sequencing data. *Genome Res.* **20**:1297–1303.

Min XJ, Butler G, Storms R, Tsang A. 2005. OrfPredictor: predicting protein-coding regions in EST-derived sequences. *Nucleic Acids Res.* **33**:W677-80.

Song L, Florea L, Langmead B. 2014. Lighter: fast and memory-efficient sequencing error correction without counting. *Genome Biol.* **15**:1.

Wheelan SJ, Church DM, Ostell JM. 2001. Spidey: a tool for mRNA-to-genomic alignments. *Genome Res.* **11**:1952–1957.

Wicker T, Mayer KF, Gundlach H, Martis M, Steuernagel B, Scholz U, Šimková H, Kubaláková M, Choulet F, Taudien S, Platzer M, *et al.* 2011. Frequent gene movement and pseudogene evolution is common to the large and complex genomes of wheat, barley, and their relatives. *Plant Cell.* **23**:1706-1718.
